# Resistance gene enrichment sequencing (RenSeq) enables reannotation of the NB-LRR gene family from sequenced plant genomes and rapid mapping of resistance loci in segregating populations

**DOI:** 10.1111/tpj.12307

**Published:** 2013-10-08

**Authors:** Florian Jupe, Kamil Witek, Walter Verweij, Jadwiga Śliwka, Leighton Pritchard, Graham J Etherington, Dan Maclean, Peter J Cock, Richard M Leggett, Glenn J Bryan, Linda Cardle, Ingo Hein, Jonathan DG Jones

**Affiliations:** 1 The Sainsbury LaboratoryNorwich Research Park, NR4 7UH, Norwich, UK; 2 The Genome Analysis CentreNorwich Research Park, NR4 7UH, Norwich, UK; 3 The Plant Breeding and Acclimatization Institute, Research Center MłochówPlatanowa 19, 05-831, Młochów, Poland; 4 Information and Computational Sciences, James Hutton InstituteDD2 5DA, Dundee, UK; 5 Cell and Molecular Sciences, James Hutton InstituteDD2 5DA, Dundee, UK

**Keywords:** NB-LRR, pathogen resistance, Solanaceae, target enrichment, next-generation sequencing, *Solanum tuberosum* Group Phureja clone DM1-3 516 R44, *Solanum ruiz-ceballosii*, *Solanum berthaultii*, *Solanum lycopersicum*, technical advance

## Abstract

**Summary:**

RenSeq is a NB-LRR (nucleotide binding-site leucine-rich repeat) gene-targeted, Resistance gene enrichment and sequencing method that enables discovery and annotation of pathogen resistance gene family members in plant genome sequences. We successfully applied RenSeq to the sequenced potato *Solanum tuberosum* clone DM, and increased the number of identified NB-LRRs from 438 to 755. The majority of these identified *R* gene loci reside in poorly or previously unannotated regions of the genome. Sequence and positional details on the 12 chromosomes have been established for 704 NB-LRRs and can be accessed through a genome browser that we provide. We compared these NB-LRR genes and the corresponding oligonucleotide baits with the highest sequence similarity and demonstrated that ∼80% sequence identity is sufficient for enrichment. Analysis of the sequenced tomato *S. lycopersicum* ‘Heinz 1706’ extended the NB-LRR complement to 394 loci. We further describe a methodology that applies RenSeq to rapidly identify molecular markers that co-segregate with a pathogen resistance trait of interest. In two independent segregating populations involving the wild *Solanum* species *S. berthaultii* (*Rpi-ber2*) and *S. ruiz-ceballosii* (*Rpi-rzc1*), we were able to apply RenSeq successfully to identify markers that co-segregate with resistance towards the late blight pathogen *Phytophthora infestans*. These SNP identification workflows were designed as easy-to-adapt Galaxy pipelines.

## Introduction

Recent advances in genome sequencing technologies have led to a dramatic reduction in costs and enable the analysis of entire crop plant genomes. Eleven years after sequencing *Arabidopsis thaliana* (The Arabidopsis Genome Initiative TAGI 2000), the genomes of two important Solanaceous crop plants, potato and tomato, were reported (Potato Genome Sequencing Consortium PGSC 2011; Tomato Genome Consortium TGC 2012). A major challenge in the post-genome sequencing era is to obtain high-quality annotations of these genomes (Yandell and Ence, 2012), so that the full potential of genome sequences can be realized for functional studies and comparative analysis (Loveland *et al*., 2012). Whole-genome sequencing of crop plants facilitates studies on genome diversity, identification of genome-wide polymorphic markers and elucidation of complex traits (Cronn *et al*., 2012). When only a subset of the genome is likely to be of interest, target enrichment offers substantial reduction in genome size and complexity prior to comparative sequencing of coding regions such as exons or specific gene families (Albert *et al*., 2007; Hodges *et al*., 2007; Cronn *et al*., 2012). Sequencing enriched samples has the advantage of yielding higher read depth for individual genes, and aids accurate identification of sequence polymorphisms in plants with large genomes and higher ploidy levels (Parla *et al*., 2011; Saintenac *et al*., 2011).

One important crop trait that is particularly suitable for target enrichment is disease resistance that is, in the vast majority of cases, conferred by dominant disease resistance (*R*) genes. R proteins typically contain nucleotide-binding (NB) and leucine-rich repeat (LRR) domains and belong to the structural class of proteins known as NB-LRRs. These can be further divided into two main groups based on their N-terminal domains and sequence motifs within the NB-ARC domain (Meyers *et al*., 1999; Dangl and Jones, 2001; Jones and Dangl, 2006; Jupe *et al*., 2012). The first group possesses a domain with homology to the intracellular signaling domains of the *Drosophila* Toll and mammalian interleukin (IL)-1 receptors and is referred to as TIR-NB-LRRs or TNLs. The second, non-TNL, group is collectively known as CC-NB-LRRs or CNLs, based on the presence of a predicted N-terminal coiled-coil domain in some, but not all, members of this class. *R* gene-based resistance acts against diseases caused by diverse and taxonomically unrelated pathogens such as bacteria, viruses, nematodes, insects, filamentous fungi and oomycetes, and is also thought to contribute to non-host resistance (Schulze-Lefert and Panstruga, 2011). In the innate plant immune system, R proteins govern a specific layer of induced resistance by recognizing, directly or indirectly, effectors from pathogens in a process known as effector triggered immunity (Jones and Dangl, 2006). NB-LRR genes are key players in plant disease resistance and their presence, absence or allelic identity is decisive for functionality.

To further understand this important gene family and to make it available for breeding purposes, significant efforts have been made to identify the NB-LRR complements from sequenced plant genomes. However, these analyses are typically limited to the available computationally predicted gene models. Within the genome of the sequenced *Solanum tuberosum* Group Phureja clone DM1-3 516 R44 (DM), a very close relative to the important crop plant potato, between 408 and 438 NB-LRR genes were predicted previously amongst the postulated 39 031 protein-coding sequences. Many were found to reside in clusters comprising closely related paralogues (PGSC 2011; Jupe *et al*., 2012). Similarly, in tomato, 356 genes were identified that contain domains typically associated with NB-LRRs (TGC 2012). The limitations of computational NB-LRR prediction in potato were described previously by Jupe *et al*. (2012) and missing domains of partially predicted genes were identified.

We present the development and applications of a NB-LRR gene-specific *R* gene enrichment and sequencing (RenSeq) workflow. Using the potato and tomato genomes as examples, we demonstrate that this method can be used to reannotate the NB-LRR gene complements from fully or partially assembled genomes using targeted resequencing. Reference gene models are only required from a single member of the target species and do not need to be complete. In addition, we have used this method for the rapid identification of molecular markers that co-segregate with resistances towards the late blight pathogen *Phytophthora infestans* in two characterized wild potato populations. We developed a two-tiered SNP-calling pipeline to identify trait-linked polymorphisms that are available as easy-to-adopt Galaxy workflows. We conclude that RenSeq provides an opportunity to quickly map functional NB-LRR-type *R* genes to control important diseases in crop plants, and a method for improvement of existing plant genome annotations.

## Results

### RenSeq in a proof-of-concept study

**Figure 1:**
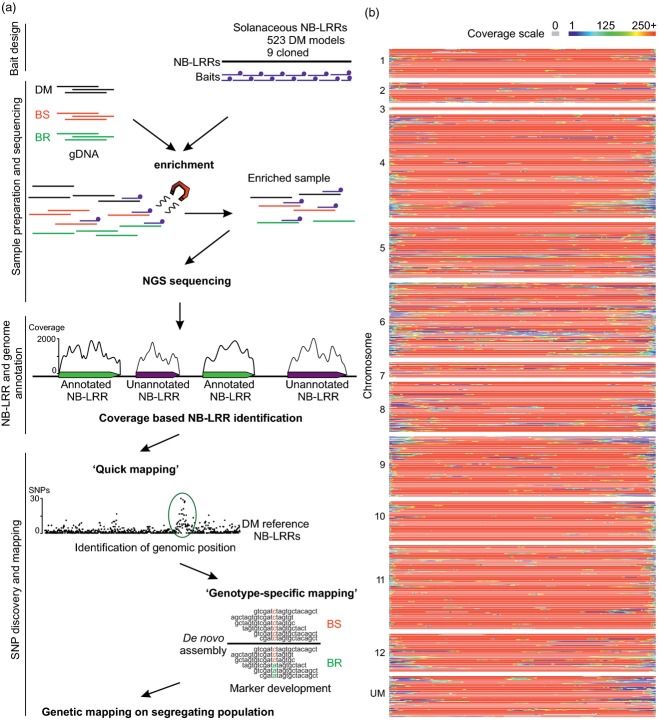
Overview of the *R* gene enrichment (RenSeq) experiment. (a) Genomic DNA from the *Solanum tuberosum* Group Phureja clone DM1-3 516 R44 was enriched for NB-LRR sequence fragments using a customized NB-LRR RNA bait-library (Agilent SureSelect). Standard Illumina PE 76 bp sequencing and BWA mapping enabled the identification of 331 additional NB-LRR loci in DM. For rapid identification of markers segregating for pathogen resistance, bulked genomic DNA of the most resistant (BR) and most susceptible (BS) phenotyped plants of segregating populations were subjected to the same workflow. Illumina reads were then applied to a ‘quick mapping’ analysis to identify the approximate chromosomal position through mapping to the 755 predicted NB-LRRs from the *Solanum tuberosum* Group Phureja DM1-3 516R44 (this work) and plotting the number of BR unique SNPs per gene. A *de novo* assembly of NB-LRR enriched reads was used as genotype-specific reference to call BR unique SNPs. These were converted into markers and used to screen the segregating population. (b) A heat map was created to visualize the read coverage over the 755 reference NB-LRR loci. Illumina sequence information for the NB-LRR enriched DM sample was mapped against the 755 NB-LRR loci from this study with default BWA settings. Each line represents one locus that was normalized to a common length. Genes are in order as on the chromosomes. UM represents unmapped genes. Colors indicate coverage, from blue (lowest) to red (highest, over 250×).

The *R* gene enrichment and sequencing (RenSeq) workflow was optimized for Illumina sequencing of genomic DNA libraries enriched for NB-LRR-encoding genes using the Agilent *SureSelect Target Enrichment System* (Agilent Technologies, USA). A customized target enrichment library comprising 48 549, 120-mer biotinylated oligos was designed based on 523 NB-LRR-like sequences that we identified from an early draft of the available potato genome annotation (PGSC 2011) and further 57 tomato NB-ARC domains and nine characterized NB-LRR-type *R* genes from tomato, tobacco and pepper (Figure 1a and Data S1).

To establish RenSeq as a genome annotation tool, the method was applied to genomic DNA of the sequenced *S. tuberosum* Group Phureja clone DM (PGSC 2011) (Figure 1a). We carried out quantitative PCR to determine the abundance of three CC-NB-LRR resistance gene homologues in DNA samples prior to and after enrichment. Normalized samples revealed approximately 290-fold enrichment (ΔΔCT of 8.15) (Table S1). We sequenced the paired-ends of fragments in these enriched libraries on an Illumina GAII platform, and assessed NB-LRR capture efficiency by high-stringency mapping of 73 million quality-controlled 76-bp reads to the DM chromosomes that contained the predicted NB-LRR coding sequences used to design the bait-library and to the previously identified 438 DM NB-LRR sequences (Jupe *et al*., 2012). After enrichment, 31.5% of all reads corresponded to the genes that featured in the bait-library design and 29.8% mapped to the 438 DM NB-LRRs (Jupe *et al*., 2012). NB-LRR gene read depth was at least 20-fold over a minimum of 500 consecutive nucleotides, and on average 4.2-fold at all other genomic regions.

To measure potential off-target enrichment, we analyzed sequence coverage of non-NB-LRR genes that featured in the negative training set used to design discriminating amino acid motifs for NB-LRR genes (Jupe *et al*., 2012). All of these genes had read coverage similar to the background, suggesting a high specificity for NB-LRR gene sequences.

### RenSeq enables reannotation of NB-LRR gene complements

**Figure 2:**
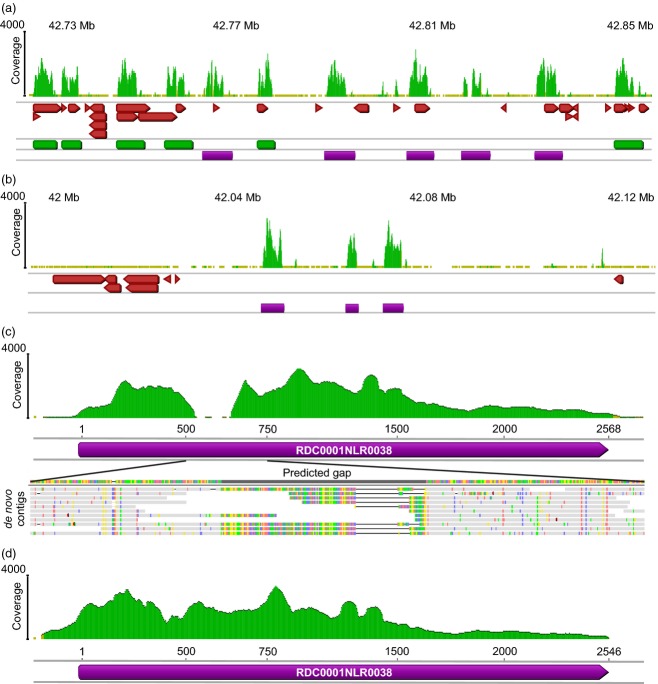
Detailed analysis of two NB-LRR gene clusters and closing of an assembly gap using RenSeq reads. The stringent mapping of RenSeq reads to the potato chromosomes and unanchored superscaffolds identified genomic regions with NB-LRR sequences. The typical read coverage over these genes is shown with green peaks. Extraction of the underlying sequences and similarity analyses improved the previously described NB-LRR gene models (green boxes; Jupe *et al*., 2012), as well as aided the discovery of additional NB-LRR loci (purple boxes) from poorly annotated (a) or non-annotated (b) regions. General PGSC predicted DM gene models are depicted as red-boxed arrows. (a) We represent a close-up of the *R3* resistance gene cluster C77, between positions 42.7Mb and 42.9Mb on chromosome 11. This analysis identified five yet uncharacterized NB-LRR loci. The identification of three NB-LRR loci from an unannotated region on chromosome 11 is depicted in (b). DM RenSeq reads and *de novo* assembled contigs were used to close gaps in the assembly of DM NB-LRRs. (c) shows the RenSeq coverage of RDC0001NLR0038 with stringently BWA mapped RenSeq reads (green peaks), and a detailed view of the alignment of *de novo* assembled contigs to the gap region. (d) The quality of the revised sequence is shown following stringent BWA mapping of RenSeq reads. Gene identifiers can be retrieved from Table S2. The figure is modified from the Geneious 5.6 genome browser view.

**Figure 3:**
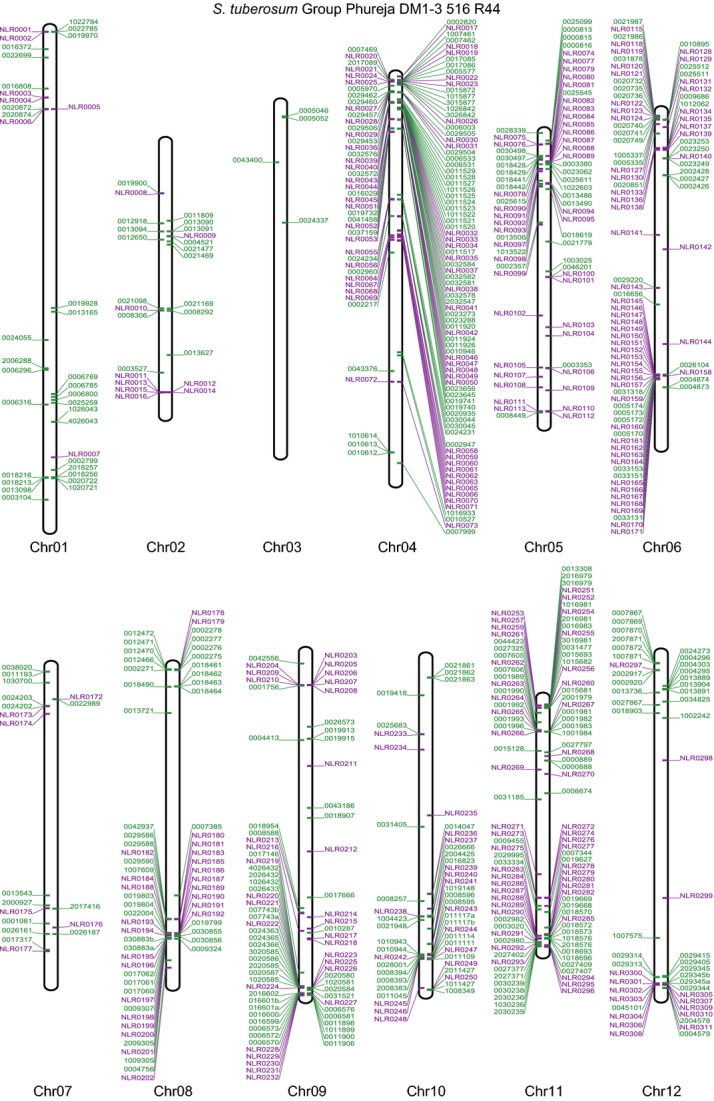
Physical mapping positions of NB-LRR loci on the 12 potato chromosomes. RenSeq of the sequenced potato clone DM as well as use of the latest potato chromosomal pseudomolecules enabled the positioning of 93% of the potato NB-LRR genes to the 12 chromosomes. The previously identified DM NB-LRR gene models (Jupe *et al*., 2012) are shown in green and those identified in this study are represented in purple. Genes to the left are on the forward strand, and to the right on the reverse strand.

Further analysis of chromosomal coverage showed that some regions within mainly unannotated sections of the genome displayed read depth patterns that could indicate the presence of NB-LRR genes (Figure 2). In total, we identified 5832 genomic fragments from the 12 DM chromosomes and unanchored superscaffolds using a cut-off of 20× RenSeq coverage over at least 500 consecutive bases as defined from the annotated NB-LRR coverage. We extracted these genomic regions and subjected them to our previously published NB-LRR identification approach using the established 20 NB-LRR-descriptive amino acid motifs in the motif alignment and search tool (MAST) to predict sequences with a motif composition similar to NB-LRR genes (Jupe *et al*., 2012; Table S2). This process identified a total of 331 yet uncharacterized NB-LRR loci, of which 304 could be placed onto the established potato chromosomes leaving 27 in currently unanchored superscaffolds (Figure 3 and Table S2). The majority (205) of the here identified NB-LRR loci resides within genomic regions for which no gene models are provided by the Potato Genome Sequencing Consortium (PGSC). Our analysis of the previously identified 438 DM NB-LRR genes (Jupe *et al*., 2012) confirmed 424 genes yielding a total of 755 NB-LRR loci in DM (Tables 1 and S2 and Data S2). Seven previously annotated NB-LRR genes were absent in the current pseudomolecule arrangement, a situation that is probably due to re-arrangements of the genome scaffolds. Further seven partial NB-LRRs were revised to yield more complete NB-LRR genes using our RenSeq data.

**Table 1:** Identification of NB-LRR loci from enriched Illumina sequencing reads

	Number of mapping DM reads^a^	Jupe *et al*., (2012)^b^	Uncharacterized sequences^c^	From unanchored DMBs^d^	NB-LRR^e^
ch01	6 313 243	28 (28)	7	1	36
ch02	3 164 710	16 (15)	8	2	25
ch03	2 722 613	4 (4)	–	–	4
ch04	9 128 545	57 (56)	50	20	126
ch05	6 359 113	27 (27)	37	4	68
ch06	6 811 178	36 (36)	48	5	89
ch07	3 114 116	13 (13)	5	1	19
ch08	5 914 143	33 (32)	14	16	62
ch09	6 618 699	44 (42)	21	12	75
ch10	6 678 663	25 (23)	16	10	49
ch11	7 845 471	54 (54)	43	6	103
ch12	4 777 140	33 (32)	16	–	48
Unanchored DMBs	5 280 770	68 (62)	66	To DMBs 77 of 128 –	51
Total	–	438 (424)	331	77	755

a Mapping of NB-LRR-enriched Illumina reads to the reference potato clone DM.

b NB-LRR genes as presented in Jupe *et al*., (2012), and confirmed in this study (in brackets if different).

c Previously uncharacterized NB-LRR encoding sequences.

d Unanchored superscaffolds (DMBs).

e Total Number of NB-LRR genes from the DM genome.

Mapping of NB-LRR-enriched Illumina reads of the reference potato clone DM aided the verification of NB-LRR genes (in brackets) as presented in Jupe *et al*., (2012) as well as the identification of previously uncharacterized NB-LRR encoding sequences. An update of the reference potato chromosomes to the latest chromosomal pseudomolecules allowed the positioning of 77 NB-LRR genes from unanchored superscaffolds (DMBs) to 10 chromosomes.

Based on the MAST analysis detailed above (Jupe *et al*., 2012), we defined the NB-LRR specific motif content for all sequences (Table S2), and identified 143 loci to harbor potential full-length NB-LRRs that contained all presumed-to-be-essential NB-ARC- and LRR-specific motifs. Using positional information of the first and last motifs, as well as predicted open reading frames, we were able to identify the putative start and stop codons for these genes (Data S2). The updated NB-LRR gene complement comprises over 2.06 Mb sequence (0.24% of the entire potato genome) assuming the previously established average length of 2.7 kb per putative gene (Jupe *et al*., 2012). Overall, 54% of the Illumina reads (39.4 million) mapped to the 755 NB-LRR loci, a finding that indicated 225-fold enrichment and corresponds well to the qPCR data. To visualize read depth, we mapped all reads to the NB-LRR complement and created a heat map that showed that most NB-LRR loci displayed a uniform coverage of at least 250-fold, with lower coverage only at the very ends (Figure 1b). All 755 NB-LRR loci were used in a blastn search (Altschul *et al*., 1990) against the previously annotated 438 genes, and revealed that 584 sequences belong to the CNL group and 157 to TNLs, while 14 sequences could not be assigned to any group.

The potato genome still contains a number of regions with unknown sequence content, and among the NB-LRR genes we identified 39 with assembly errors presented as stretches of Ns of varying length. We randomly selected nine NB-LRR genes, and mapped *de novo* Velvet-assembled contigs of the DM RenSeq reads to their corresponding reference sequence under relaxed conditions. Sequence information spanning the entire gap regions allowed us to revise the length of these ambiguous fragments and we were able to close three gaps of up to 141 nt length (DMG 0032578, RDC0001NLR0038 and RDC0 001NLR0327). As an example, RDC0001NLR0038 is shown in Figure 2(c,d). The results were confirmed by very stringent mapping of DM RenSeq reads, with polymorphism free sequence overhangs into available flanking regions. For further five putative NB-LRRs (RDC0001NLR0027, RDC0001NLR0286, DMG 0022785, DMG 2018576 and DMG 0007869), assembled contigs were not long enough to span the entire gap and, therefore, up to four rounds of iterative mapping of RenSeq reads followed by narrowing the gap using the overhanging sequencing reads was conducted. In the case of RDC0001NLR0295, we were unable to resolve the gap even after four rounds of mapping and iterative closing, and believe a genomic misassembly could be the cause. By mapping *de novo* assembled contigs, we were further able to identify and revise three deletions in two sequences (seven nt in DMG 0032578 and 35/41 nt in RDC0001NLR0027), which we again confirmed by stringent mapping of paired-end RenSeq reads.

Using the latest available DM chromosomal pseudomolecules version 4.03 (Potato Genome Mapping Group; http://solanaceae.plantbiology.msu.edu/), we could position 704 NB-LRR loci (93%) on the 12 chromosomes, whilst 51 loci (6.7%) remain on currently unanchored superscaffolds. The current potato chromosomal pseudomolecules showed significant changes with respect to those used in our earlier analysis (Jupe *et al*., 2012), including inversions and translocations of chromosomal segments (Potato Genome Mapping Group, personal communication). Revised chromosomal locations for all NB-LRRs are depicted in Figure 3 and in more detail in Figure S1. We further created a DM NB-LRR specific genome browser with full access to sequence and positional information (http://solanum.hutton.ac.uk). In line with previous data (Jupe *et al*., 2012), 76% (577) of the chromosomal anchored NB-LRR loci reside within a total of 92 clusters (Tables S2 and S3). Next to the expansion of existing clusters (Figure 2a), we observed the emergence of, among others, a CNL cluster on chromosome 11 (NLR0278 to NLR0280; three members; Figure 2b), and two TIR-NB-LRR clusters on chromosome 6 (NLR0145 to NLR0159; 17 members) and chromosome 12 (NLR0300 to NLR0310; 12 members; Figure S1 and Table S2), respectively.

### Theoretical prediction of the tomato NB-LRR complement

We have shown that the RenSeq experiment and analysis significantly expanded the existing potato NB-LRR gene complement. To establish whether our bait-library could improve the annotation of the second sequenced important *Solanum* crop tomato, we applied an *in silico* version of this approach to the available tomato NB-LRR annotation. The tolerance for sequence diversity between baits and target genes was established bioinformatically through local alignments. We applied the blastn algorithm to search for sequence similarities between the reads and the bait-library, and produced a distribution of sequence identities. We included in this analysis RenSeq data that we obtained by NB-LRR-enrichment and Illumina sequencing from the reference DM and four diverse *Solanum* species, three potato (*S. ruiz-ceballosii, S. berthaultii, S. michoacanum*) and a nightshade (*S. nigrum*). The lowest sequence identity to any bait that we found *in silico* amongst the sequenced reads was 80.3%. This analysis, however, does not take into account any chemical or physical properties that would allow for more mismatches to occur during the hybridisation between RNA-baits and DNA fragments.

**Figure 4:**
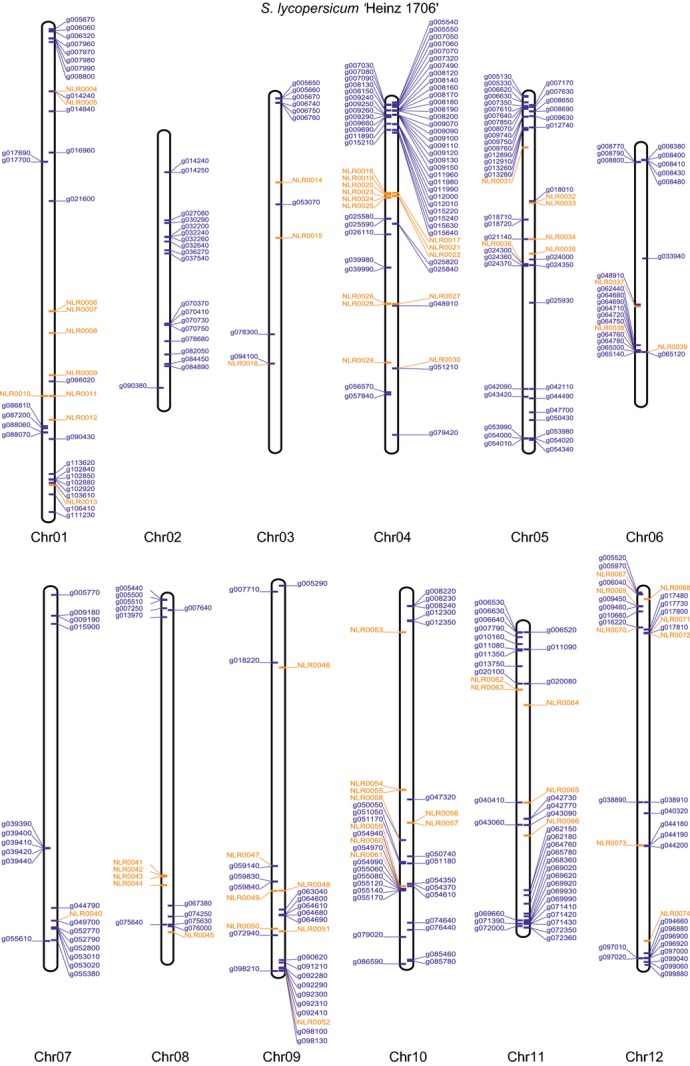
Physical mapping positions of NB-LRR loci on the 12 tomato chromosomes. An empirically determined threshold of 80% sequence identity between bait and DNA fragments was used to screen for sequence stretches on the assembled tomato chromosomes with similarity to sequences in the bait library. This analysis identified 394 tomato NB-LRR loci, of which 387 could be positioned to the 12 chromosomes. The previously reported tomato NB-LRR loci (TGC 2012) are shown in blue and those identified in this study in orange. Genes to the left are on the forward strand, and to the right on the reverse strand.

We used this empirically determined threshold to screen for sequence stretches within the assembled tomato chromosomes (TGC 2012) *in silico* that contained bait matches with at least 80% sequence identity. An analysis of randomly selected tomato NB-LRRs (TGC 2012) determined that at least four unique baits aligned to their predicted sequence. Applying these criteria, 531 regions were extracted including up to 2kb flanking regions and we subsequently analyzed these regions using blastn searches against the DM NB-LRR set and NCBI nr-database. This identified an additional 67 yet uncharacterized NB-LRR encoding regions and, similar to potato, most of these were within previously unannotated regions. For 29 tomato gene models that were annotated as NB-LRRs by the TGC, the sequence search did not retrieve any NB-LRR-like hit. Additional blastn searches against the NCBI nr-database also failed to confirm sequence similarity to NB-LRR genes and let us conclude that these are most likely annotation errors. Further sequence similarity searches suggested that several physically adjacent NB-LRR fragments could be combined to form complete coding sequences. In total, we identified 394 NB-LRR encoding loci from the tomato genome, and we were able to position 387 on the 12 chromosomes (Figure 4 and Table S4). This analysis provides a further application for the DM NB-LRR bait-library, to *in silico* predict the NB-LRR gene complements from other sequenced Solanaceae genomes.

### Bulked segregant analysis for linked marker development in *Solanum* species

We successfully utilized RenSeq to enrich for NB-LRRs from two segregating wild potato populations to identify SNPs within NB-LRR gene sequences that are directly linked with pathogen resistance (Figure 1a). We used two established populations segregating for resistance towards the late blight pathogen *P. infestans*, derived from the outcrossing species *Solanum berthaultii* (*Rpi-ber2*; Park *et al*., 2009) and *Solanum ruiz-ceballosii* (*Rpi-rzc1*; Śliwka *et al*., 2012). The underlying single dominant *R* genes *Rpi-ber2* and *Rpi-rzc1* have previously been mapped in F1 segregating populations to the long arm of chromosome 10, where several NB-LRR gene clusters are present (Park *et al*., 2009; Bakker *et al*., 2011; Jupe *et al*., 2012 and this work). Due to the outcrossing nature of these *Solanum* spp., the resistant parents are heterozygous at the resistance locus and the resulting F1-population segregates 1:1 for pathogen recognition. Prior to enrichment, we bulked susceptible (BS) and resistant (BR) gDNA samples (25 individuals for *Rpi-ber2*, 50 individuals for *Rpi-rzc1*) and sequenced post-enrichment samples on an Illumina GAII (Figure 1a). A qPCR was carried out as described above and revealed an enrichment of NB-LRR sequences of ∼300-fold.

We further analyzed the enrichment efficiency and read depth over the established NB-LRR loci as described above. Similar to the high uniformity that we found for the DM enrichment (Figure 1a), the read depth coverage for most NB-LRRs from *S. berthaultii* and *S. ruiz-ceballosii* was above 250-fold which reduced to below 100-fold in the 5′ and 3′ ends (no single reads allowed, Figure S2). This confirms the ability to apply the bait-library-based NB-LRR gene enrichment to a divergent set of *Solanum* species at high efficiency.

### ‘Quick mapping’ to the DM NB-LRR reference

**Figure 5:**
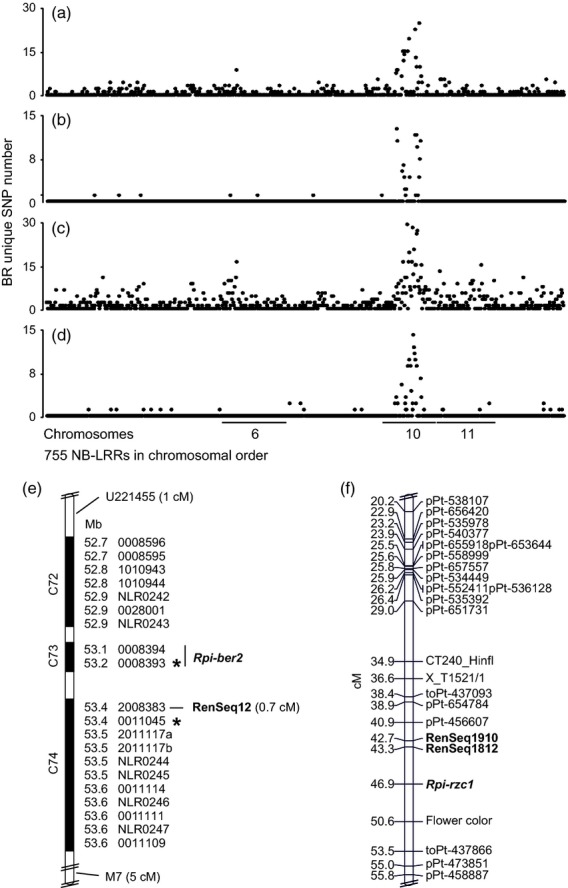
‘Quick’ and ‘genotype-specific mapping’ analyses identify *R* gene candidate loci in *S. berthaultii* and *S. ruiz-ceballosii*. ‘Quick mapping’ (a,c) was carried out for NB-LRR enriched Illumina reads of BS and BR DNA of *S. berthaultii* and *S. ruiz-ceballosii* segregating populations to the 755 DM NB-LRR loci followed by SNP calling. The number of SNPs is plotted over the 755 NB-LRR sequences in chromosomal order. A high peak above chromosome 10 represents an enrichment of BR specific sequence variations that was used to identify the approximate candidate NB-LRR loci. Subsequent *de novo* assembly and ‘genotype-specific mapping’ to the resulting contigs (b,d) further reduced the background of SNPs and allowed a more specific resolution of variations that are unique to the BR individuals. These variations were further used to create genetic markers that were used to analyze the segregating populations. Genotyping the populations resulted in a physical linkage map (e) for the *Rpi-ber2* population with closely linked markers derived from NB-LRR homologues within clusters C73 and C74 (Table S2). RenSeq12 depicts the closest marker linked to resistance, which was identified in this study. U221455 and M7 depict the closest markers that were previously identified using standard mapping methods (Rauscher *et al*., 2006, WV). Physical positions of NB-LRR genes are given in Mb, and are based on the reference DM genome (Table S2). Asterisks represent the position of additional Sanger confirmed but not genetically mapped markers. A genetic linkage map (f) of the *S. ruiz-ceballosii* chromosome 10 shows the location of the late blight resistance gene (*Rpi-rzc1*), and two groups of close RenSeq markers represented by RenSeq1910 and RenSeq 1812. Cumulative genetic distances are shown in cM.

We developed a two-tiered SNP-calling pipeline, using two reference genomes (Figure 1a). During the first ‘quick mapping’, we utilize the existing DM NB-LRR loci sequences as a reference to identify genomic regions enriched for sequence variations. For both diploid populations we separately mapped BS and BR paired-end reads using BWA (Li and Durban, 2009). Based on the genetically dominant nature of the mapped resistance, susceptible plants are expected to be homozygous at the functional resistance gene locus and resistant plants heterozygous, with an expected ratio of 1:1. However, our pipeline can be adjusted to detect SNPs in populations with other predicted ratios. To facilitate the analysis of short Illumina reads of very similar allelic and paralogous NB-LRRs, we searched for BR specific polymorphisms, where the alternate base was present in at least 25% of the reads at this position and ‘absent’ in BS (cut-off 5%), and plotted the numbers of predicted SNPs for each of the 755 DM NB-LRR reference sequences (Figure 5a,c). This mapping confirmed the previously reported position of both genes, *Rpi-ber2* and *Rpi-rzc1,* within the NB-LRR gene clusters C71 to C74. Therefore, this presents an important application of RenSeq and the re-annotated NB-LRR complement of DM to define more rapidly candidate resistant loci.

### ‘Genotype-specific mapping’ based on a *de novo* NB-LRR assembly

The ‘quick mapping’ identified the potential locus as well as candidate genes based on the sequenced reference DM. However, this does not allow the prediction of the NB-LRR complement and its organization in other *Solanum* species and genotypes. We therefore assembled reads from BS DNA to generate a genotype-specific reference sequence set (Figure 1a). Using Velvet (Zerbino and Birney, 2008), all left reads passing quality control of *S. berthaultii* and *S. ruiz-ceballosii* were assembled, producing 4368 and 4762 contigs for *S. berthaultii* and *S. ruiz-ceballosii* respectively, with a median length above 200nt and an N50 of 429 and 290, respectively (Table S5). Using MUMmer (Kurtz *et al*., 2004) we aligned 1303 and 1932 of the contigs to our reference DM NB-LRR set for *S. berthaultii* and *S. ruiz-ceballosii*, respectively, covering 67% and 70% of the DM NB-LRR loci with at least one contig over more than 5% of the sequence length. NB-LRRs situated on chromosome 10 exhibited a higher coverage in both species with 82% and 92%, respectively (Figure S3). Sanger sequencing of selected contigs revealed that more than 70% of the contigs were properly assembled.

We used this *de novo* assembly as a reference to call sequence variants between BR and BS, and to calculate the number of variants per DM reference NB-LRR. This number was compared to the number of SNPs derived from the initial ‘quick’ mapping (Figure 5), confirming the previously ‘quickly’ established results, of members of the yet uncharacterized CNL-7 family from four clusters on chromosome 10 (C71 to C74; Jupe *et al*., 2012). As expected, ‘genotype-specific mapping’ produces a lower background due to the reference used, and in the case of *S. ruiz-ceballosii* removed two additional peaks from chromosomes 6 and 11 (Figure 5c,d).

### Genetic mapping of *de novo* assembly derived markers

To confirm the bioinformatically inferred positions of the two *Rpi* genes, we used the above predicted polymorphisms within NB-LRRs to develop PCR markers around SNPs that showed approximate 1:1 segregation of the two alleles. We identified 26 and 29 contigs, some with multiple SNPs, for *S. berthaultii* and *S. ruiz-ceballosii*, respectively (Table S6). Out of these contigs, 10 from *S. berthaultii* and four contigs from *S. ruiz-ceballosii* could not unambiguously be positioned to chromosome 10 and Sanger sequencing confirmed that these were false positives. However, based on our previous results from the ‘quick’ and ‘genotype-specific mapping’ these would have been ignored in subsequent analyses. The remaining 16 and 25 *S. berthaultii* and *S. ruiz-ceballosii* contigs clustered on chromosome 10 (Figure 5) and were further amplified from the BR and BS genomic DNA pools. Sanger sequencing of these PCR products confirmed predicted SNPs in seven *S. berthaultii* and 10 *S. ruiz-ceballosii* contigs that could be anchored to the *Rpi-ber2* and *Rpi-rzc1* NB-LRR cluster on chromosome 10 (belonging to clusters C71 to C74; Park *et al*., 2009; Jupe *et al*., 2012). The predicted nucleotide variation had a false-positive ratio of 31% (*S. berthaultii*) and 8% (*S. ruiz-ceballosii*), and the remaining contigs contained either multiple reads or double peaks due to amplification of similar sequences.

To test the robustness of our methods, we fine mapped *Rpi-ber2* by screening 1187 F1 plants for recombination between CAPS markers U221455 (Rauscher *et al*., 2006) and M7 (a marker proximal to *Rpi-ber2*, WV) and found 68 recombinants, which were subsequently phenotyped and further genotyped using a RenSeq-derived CAPS marker (‘genotype-specific mapping’) in DMG 2008383. This analysis identified eight recombinants, positioning this marker at 0.7 cM from *Rpi-ber2* (Figure 5).

To genetically position the *Rpi-rzc1* RenSeq-specific markers, we used a segregating population (Śliwka *et al*., 2012) and showed that they mapped into two groups, at 3.6 cM and 4.2 cM from the *Rpi* gene. The groups of each four markers are represented as RenSeq1812 and RenSeq1910 in the genetic map (Figure 5).

These results show that the initial ‘quick mapping’ to the DM NB-LRR sequences identifies the target chromosome and candidate NB-LRR cluster, while subsequent ‘genotype-specific mapping’ based on *de novo* assembly can rapidly produce closely linked markers which can be used for fine mapping of the resistance allele.

## Discussion

In this paper, we present RenSeq as a refined method that enables us to capture and sequence the whole NB-LRR-type *R* gene complement from various *Solanum* spp. Moreover, we show that RenSeq can be used to improve existing NB-LRR gene annotations and reveal NB-LRR loci from uncharacterized genomes of Solanaceous species, using potato and tomato as examples. In contrast to existing NGS-based genotyping techniques, this *R* gene capture method reduces the genome complexity in a non-random manner to preferentially isolate the target gene family. In conjunction with a bulked segregant analysis (BSA), this approach allows us to detect polymorphism co-segregating with, or closely linked to, functional *R* gene alleles.

The various analyses of NB-LRR genes of potato and tomato described so far (Jupe *et al*., 2012; Lozano *et al*., 2012; Andolfo *et al*., 2013) were solely based on the existing automated gene and protein predictions of the Potato and Tomato Genome Sequencing efforts (PGSC 2011; TGC 2012). Here, we used experimentally derived sequence data to identify 331 previously unannotated NB-LRR sequences in the potato clone DM. Furthermore, by mapping the bait-library sequences under experimentally verified stringencies to the tomato genome, we were able to verify current NB-LRR gene models, eliminate wrong annotations and reannotate existing models. The NB-LRR gene complements for both important crops have increased to 755 in DM and 394 in tomato, revealing further NB-LRR clusters. Of the genes described in this study, 75% reside in poorly or previously unannotated regions of the potato and tomato genome.

The limitation of gene or gene fragment identification requires alternative approaches to annotate gene families of interest. We previously demonstrated that many NB-LRR genes that were annotated as partial (PGSC 2011) were actually full length, with the missing sequences residing in the flanking regions (Jupe *et al*., 2012). Many of the NB-LRRs that are identified and presented in this study were found to reside within previously identified clusters of genes (Figure 2). Furthermore, most genes are putatively full length and many have putative RNAseq coverage (http://solanaceae.plantbiology.msu.edu/cgi-bin/gbrowse/potato/). There is thus no obvious reason arising from the sequence information why the gene prediction software failed to annotate them. That we were able to identify these genes validates RenSeq as an annotation tool.

NB-LRR gene sequences are highly conserved among plant species, and thus RenSeq relies initially on a partially annotated genome of a relative to the species of interest to design the bait-library. Therefore, without relying on gene models of poorly or unannotated genomes, RenSeq provides a valid tool to discover NB-LRR loci rapidly. The sequence similarity among NB-LRR genes enables deep coverage over all NB-LRR domains. These regions can be verified further using our NB-LRR gene-specific MEME motifs and MAST search pipeline (Jupe *et al*., 2012) and, if necessary, annotated through further predictions of open reading frames or through alignments to other NB-LRR sequences.

RenSeq-derived sequence data allow not only the identification of the NB-LRR gene complements, but also the revision of gene models provided by the sequencing consortia of the respective organisms. We provide examples of RenSeq data being used to close gaps, identify deletions within NB-LRR sequences of the DM reference genome and to correct misassembled sequences. This approach is especially relevant for heterozygous species, including most wild potato relatives.

Here we show that short RenSeq Illumina reads can be assembled *de novo* with high accuracy to generate contigs, representing the majority of the NB-LRR loci. Although these assemblies in most cases do not span whole gene models, they can still be used as genotype-specific references to study NB-LRRs across species or genotypes. This approach eliminates the necessity to sequence whole genomes, and allows also the exploration of allelic diversity. We show that BSA combined with targeted short read sequencing of NB-LRR sequences enables SNP calling within members of this gene family, using a *de novo* assembly of BSA derived RenSeq data as a reference. The presented computational analysis enables the identification of NB-LRR alleles that co-segregate with the underlying resistance in hitherto unsequenced species. In comparison with DArT, another high-throughput genotyping technique (Jaccoud *et al*., 2001), RenSeq aided the development of the closest markers for the *Rpi-rzc1* resistance (Śliwka *et al*., 2012) within a cluster of candidate resistance genes.

One drawback that we encountered is that although all NB-LRR genes are highly covered with RenSeq reads it is difficult to assemble full-length genes with a high degree of confidence from Illumina 76-bp sequences. This situation is mainly due to the number of large gene sub-families and the high sequence similarity between paralogs and alleles. However, emerging sequencing technologies that are compatible with RenSeq such as single molecule PacBio RS (Pacific Bioscience, Ltd.) sequencing, which can potentially yield 20 kb long DNA fragments, provide suitable alternatives for overcoming this current limitation of the approach.

The here presented method can be designed for, and applied to, any multigene family, both to improve the annotation and to identify polymorphisms between parental lines and populations that segregate for a specific trait.

## Experimental Procedures

### Design of a customized NB-LRR enrichment library

Capture of NB-LRR like sequences was carried out using a solution-based customized Agilent SureSelect Target Enrichment kit (Agilent Technologies Inc., Santa Clara, CA, USA), using the first draft of the potato NB-LRR gene complement. For further details see Methods S1.

### Plant material and DNA extraction

The F1 populations used in this study have been described previously (*Rpi-ber2;* Park *et al*., 2009); *Rpi-rzc1* (Śliwka *et al*., 2012). The sequenced *Solanum tuberosum* Group Phureja clone DM1-3 516 R44 (DM) was described in PGSC (2011). Extraction of genomic DNA for the RenSeq approach was carried out using a CTAB protocol (Weigel, 2002) and for the F1 recombinant screen using a 10% Chelex solution (BioRad, Hercules, CA, USA). Equimolar amounts of DNA were pooled from 25 susceptible and resistant plants of the *Rpi-ber2* population, and 50 plants of the *Rpi-rzc1* population, respectively.

### Target capture

Target capture was carried out in accordance with the Agilent protocol but with minor modifications. For further details see Methods S1.

### Illumina sequencing and raw sequence data processing

All quality control, mapping and sequence assembly experiments of Illumina Inc. (San Diego, CA, USA) paired-end sequences were carried out using tools embedded in The Sainsbury Laboratory (TSL) customized Galaxy instance (Giardine *et al*., 2005; Blankenberg *et al*., 2010; Goecks *et al*., 2010; Maclean and Kamoun, 2012), if not noted otherwise. For further details see Methods S1.

### Coverage picture

QC reads of DM and BS of *Rpi-ber2* and *Rpi-rzc1* were mapped to the 755 DM NB-LRR sequences using BWA with default parameters and a pileup file was created using BWA's MPileup command (Li and Durban, 2009). From these data, a separate file of coverage information for each position in each gene was extracted. A custom Java application was used to convert this file into a heat map image, using the same scale for each gene and further normalizing by stretching each gene to fill the full width of the image.

### Identification and annotation of DM NB-LRR loci

To identify and annotate NB-LRR loci that are not present in the PGSC provided gene models (PGSC 2011), NB-LRR enriched paired-end Illumina reads were mapped to the 12 pseudomolecules and unanchored superscaffolds individually, using BWA (default parameters) (PGSC_DM_ST4.03.fasta PGSC_DM_v3_2.1.9_superscaffolds_unanchored_gtr_2.5k.fasta). The mapping information (BAM-format) was imported into Geneious and visualized on the single chromosomes. The Illumina read coverage over previously identified NB-LRRs was determined for superscaffold PGSC0003DMB000000008 as a valid test sequence, for which a minimum coverage of 25 over at least 550 consecutive nucleotides was determined. Regions harboring potential NB-LRR loci were selected using more inclusive parameters of minimum 20× coverage over minimum 500 nt length.

All retrieved potential NB-LRR harboring loci were subjected to a blastn search against the 438 DM NB-LRR genes (Jupe *et al*., 2012), and if no hit was retrieved, against the NCBI nr-database. Sequences with NB-LRR specific hits were further queried in a MAST (Bailey and Elkan, 1994) search as described in Jupe *et al*. (2012), and a BLAT (Kent, 2002) search against the latest version of the chromosomal pseudomolecules and corresponding unanchored superscaffolds determined the position of the candidate sequences. The derived positions were checked for overlap with the 438 DM NB-LRRs.

Potential full-length sequences were determined using the MAST output as described in Jupe *et al*. (2012) (Table S2). These genes harbor potentially all NB-ARC and LRR specific motifs associated with functional genes. The positional information of these motifs together with the open reading frame finding function in Geneious guided the identification of the potential start and stop codons.

Gaps in the assembly were closed by initially mapping *de novo* assembled contigs in Geneious. Contiguous nucleotides overlapping into the gap region were added to the reference and this was used to BWA map all DM RenSeq reads under conditions that allow a maximum of 1% mismatches. Mapping of the *de novo* assembled contigs also identified a number of potential deletions in the reference. Mapping results around gaps and/or deletions were visualized in Geneious, and only nucleotides of reads without polymorphism on the available reference sequence were considered for further closing of the gaps. Mapping and manual iteration were repeated up to four times. Only reads without polymorphism in the aligning region to the reference were chosen for further closing the gaps.

Chromosomal positions (Figures 4 and S1) of all mapped potato and tomato NB-LRR loci were visualized using a custom Biopython script (Cock *et al*., 2009; Jupe *et al*., 2012).

For future reference, we decided to name the discovered potential *R* gene loci, which include all NB-LRRs, as follows: RDC0001NLR0001, in which *RDC* stands for *R gene discovery consortium*, the following four digits are reserved for the analyzed plant species, and NLR0001 identifies the corresponding NB-LRR.

All reads were mapped to the here described NB-LRR set of 755 sequences, and the number of mapping reads per reference sequence was determined using SAM-tools (Li and Durban, 2009).

### Applying the bait-library to reanalyze tomato NB-LRR gene models

To determine the maximum distance between bait and captured target, we established the theoretical distance bioinformatically through local alignments. We cannot rule out that baits with lower identity than 80.3% facilitated the enrichment. The established value was used to improve the NB-LRR annotation in tomato. For further details see Methods S1.

### ‘Quick’ and ‘Genotype-specific mapping’

The ‘quick’ and ‘genotype-specific mapping’ approaches were carried out using tools included in the TSL customized Galaxy instance if not noted otherwise. The Galaxy workflows are made available as Methods S2 for ‘quick mapping’, and Methods S3 for ‘genotype-specific mapping’. For ‘quick mapping’ quality controlled (as described above) BS and BR PE reads were mapped separately to 755 NB-LRRs (this work) using BWA with default settings. The resulting SAM files were filtered for mapped reads and PCR/optical duplicates, and further converted into BAM files. An MPileup file was created from the BAM information and subsequently filtered for base quality (>20) and nucleotide coverage of higher than 50. The percentage of the variant allele was calculated for each position and those with an alternative allele >25% in BR and <5% in BS were used further. Next, BR and BS positions matching the above criteria were compared and common ones kept as BR unique SNPs. The number of SNPs per NB-LRR reference sequence was calculated and plotted in chromosomal order using R (R Core Developers, http://www.r-project.org). Sequences for which no polymorphism was detected received a value 0 for the graph.

‘Genotype-specific’ mapping was carried out similarly with minor changes. *De novo* assembled contigs of left BS reads (see below) were used as a reference and as the median contig length was shorter than the insert size used for the library construction (600 nt), we mapped left and right reads separately and performed SNP calling (as described above). Polymorphic positions in each contig were compared between left and right reads and common variations used as BR unique SNPs. The total number of SNPs was calculated for each contig. Using MUMmer (Kurtz *et al*., 2004) all contigs were aligned against 755 DM NB-LRRs allowing for 10% gaps and mismatches and based on this alignment, the total number of SNPs was calculated per each DM NB-LRR gene, and the results were plotted in R as described above.

### *De novo* assembly

After running VelvetOptimiser with default settings, using k-mer length from 41 to 69, DM, *Rpi-ber2* and *Rpi-rzc1* BS QC left and right reads were assembled with a k-mer length of 57. Larger numbers of contigs with higher n50 value were obtained from the left reads for both populations, and therefore used for further analysis. The Galaxy assembly statistics tool was used to calculate the n50 value and the remaining statistical data (Table S5)

### Design of genetic markers, amplification and genetic mapping

Contigs with a ratio 1:1 (or close to) of reference to alternative nucleotide in BR, and close to 0% of the alternative nucleotide in BS were selected, and the Illumina read mapping information was displayed and verified manually using the Savant Genome Browser (http://www.genomesavant.com). Primers that flank the predicted polymorphic positions in the selected contigs (Table S6) were designed using VectorNTI (Life Technologies, Carlsbad, CA, USA) and amplified from BS and BR gDNA in 25-μl PCR reactions (35 cycles at 58°C), using standard laboratory *Taq* polymerase. Samples that contained single PCR bands were Sanger sequenced at TGAC (Norwich, UK), and analyzed using VectorNTI software.

Designed CAPS markers were amplified from individual plants of the segregating populations as above, and digested with the appropriate restriction enzyme (New England Biolabs, Inc., Ipswich, MA, USA) for 2 h at the required temperature. Digestion products were visualized on 1.5% agarose gels.

### Linkage map construction

For *Rpi-rzc1*, linkage analyses were performed using JoinMap® 4 (Van Ooijen, 2006) with the following settings: Cross Pollinating population type (CP), independence LOD as a grouping parameter (linkages with LOD >3 were considered significant), regression mapping algorithm and Kosambi mapping function. *Rpi-ber1* map was constructed manually, using the corresponding marker positions on the reference DM and NB-LRR positions, assuming one recombination per 100 plants equals one cM.

### Data access

All described NB-LRR enriched Illumina sequence reads were deposited as raw data at the European Nucleotide Archive under the following accession number ERP002644.

## SUPPORTING INFORMATION

Figure S1 Physical mapping position of all potato NB-LRR loci.

Figure S2 Visualisation of NB-LRR read coverage after RenSeq of DM, *Rpi-ber2* and *Rpi-rzc1.*

Figure S3 The coverage of chromosome 10 DM NB-LRRs with *de novo* assembled contigs from *Rpi-ber2* and *Rpi-rzc1*.

Table S1 Assessment of NB-LRR enrichment efficiency using quantitative PCR.

Table S2 List of identified DM potato NB-LRR loci including position, strand and MAST search result.

Table S3 Number of clustered NB-LRR loci and number of clusters per chromosome.

Table S4 List of identified tomato NB-LRR loci.

Table S5 Statistics of Velvet *de novo* assembly of RenSeq reads.

Table S6 Primer list and Sanger sequencing results for markers designed over potential polymorphisms that co-segregate with resistance.

Table S7 Primer sequences used in a qPCR to study the enrichment efficiency of NB-LRR sequences.

Data S1 The bait-library sequences.

Data S2 A FASTA-file for the 755 DM NB-LRR loci sequences.

Methods S1 A detailed section containing all experimental procedures.

Methods S2 Galaxy workflow file for the 'quick mapping' approach designed in this study.

Methods S3 Galaxy workflow file for the 'genotype-specific mapping' approach designed in this study.

## References

[b1] Albert TJ, Molla MN, Muzny DM (2007). Direct selection of human genomic loci by microarray hybridization. Nat. Methods.

[b2] Altschul SF, Gish W, Miller W, Myers EW, Lipman DJ (1990). Basic local alignment search tool. J. Mol. Biol.

[b3] Andolfo G, Sanseverino W, Rombauts S, Van de Peer Y, Bradeen JM, Carputo D, Frusciante L, Ercolano MR (2013). Overview of tomato (Solanum lycopersicum) candidate pathogen recognition genes reveals important Solanum R locus dynamics. New Phytol.

[b4] Bailey TL, Elkan C (1994). Fitting a mixture model by expectation maximization to discover motifs in biopolymers. Proc. Int. Conf. Intell. Syst. Mol. Biol.

[b5] Bakker E, Borm T, Prins P (2011). A genome-wide genetic map of NB-LRR disease resistance loci in potato. Theor. Appl. Genet.

[b6] Blankenberg D, Von Kuster G, Coraor N, Ananda G, Lazarus R, Mangan M, Nekrutenko A, Taylor J (2010). Galaxy: a web-based genome analysis tool for experimentalists. Curr. Protoc. Mol. Biol.

[b7] Brommonschenkel SH, Frary A, Tanksley SD (2000). The broad-spectrum tospovirus resistance gene Sw-5 of tomato is a homolog of the root-knot nematode resistance gene Mi. Mol. Plant- Microbe Interact.

[b8] Cock PJ, Antao T, Chang JT (2009). Biopython: freely available Python tools for computational molecular biology and bioinformatics. Bioinformatics.

[b9] Cronn R, Knaus BJ, Liston A, Maughan PJ, Parks M, Syring JV, Udall J (2012). Targeted enrichment strategies for next-generation plant biology. Am. J. Bot.

[b10] Dangl JL, Jones JD (2001). Plant pathogens and integrated defence responses to infection. Nature.

[b11] Ernst K, Kumar A, Kriseleit D, Kloos DU, Phillips MS, Ganal MW (2002). The broad-spectrum potato cyst nematode resistance gene (Hero) from tomato is the only member of a large gene family of NBS-LRR genes with an unusual amino acid repeat in the LRR region. Plant J.

[b12] Fiume M, Smith EJ, Brook A, Strbenac D, Turner B, Mezlini AM, Robinson MD, Wodak SJ, Brudno M (2012). Savant Genome Browser 2: visualization and analysis for population-scale genomics. Nucleic Acids Res.

[b13] Giardine B, Riemer C, Hardison RC (2005). Galaxy: a platform for interactive large-scale genome analysis. Genome Res.

[b14] Goecks J, Nekrutenko A, Taylor J (2010). Galaxy: a comprehensive approach for supporting accessible, reproducible, and transparent computational research in the life sciences. Genome Biol.

[b15] Hodges E, Xuan Z, Balija V (2007). Genome-wide in situ exon capture for selective resequencing. Nat. Genet.

[b16] Ishibashi K, Masuda K, Naito S, Meshi T, Ishikawa M (2007). An inhibitor of viral RNA replication is encoded by a plant resistance gene. Proc. Natl Acad. Sci. U.S.A.

[b17] Jaccoud D, Peng K, Feinstein D, Kilian A (2001). Diversity arrays: a solid state technology for sequence information independent genotyping. Nucleic Acids Res.

[b18] Jones JD, Dangl JL (2006). The plant immune system. Nature.

[b19] Jupe F, Pritchard L, Etherington GJ (2012). Identification and localisation of the NB-LRR gene family within the potato genome. BMC Genomics.

[b20] Kent WJ (2002). BLAT—the BLAST-like alignment tool. Genome Res.

[b21] Kurtz S, Phillippy A, Delcher AL, Smoot M, Shumway M, Antonescu C, Salzberg SL (2004). Versatile and open software for comparing large genomes. Genome Biol.

[b22] Lanfermeijer FC, Dijkhuis J, Sturre MJ, Haan de P, Hille J (2003). Cloning and characterization of the durable tomato mosaic virus resistance gene Tm-2(2) from Lycopersicon esculentum. Plant Mol. Biol.

[b23] Li H, Durban R (2009). Fast and accurate short read alignment with Burrows-Wheeler transform. Bioinformatics.

[b24] Loveland JE, Gilbert JG, Griffiths E, Harrow JL (2012). Community gene annotation in practice. Database (Oxford).

[b25] Lozano R, Ponce O, Ramirez M, Mostajo N, Orjeda G (2012). Genome-wide identification and mapping of NBS-encoding resistance genes in Solanum tuberosum Group Phureja. PLoS One.

[b26] Maclean D, Kamoun S (2012). Big data in small places. Nat. Biotechnol.

[b27] Meyers BC, Dickerman AW, Michelmore RW, Sivaramakrishnan S, Sobral BW, Young ND (1999). Plant disease resistance genes encode members of an ancient and diverse protein family within the nucleotide-binding superfamily. Plant J.

[b28] Milligan SB, Bodeau J, Yaghoobi J, Kaloshian I, Zabel P, Williamson VM (1998). The root knot nematode resistance gene Mi from tomato is a member of the leucine zipper, nucleotide binding, leucine-rich repeat family of plant genes. Plant Cell.

[b29] Ori N, Eshed Y, Paran I, Presting G, Aviv D, Tanksley S, Zamir D, Fluhr R (1997). The I2C family from the wilt disease resistance locus I2 belongs to the nucleotide binding, leucine-rich repeat superfamily of plant resistance genes. Plant Cell.

[b30] Park T-H, Foster SJ, Brigneti G, Jones JDG (2009). Two distinct potato late blight resistance genes from Solanum berthaultii are located on chromosome 10. Euphytica.

[b31] Parla JS, Iossifov I, Grabill I, Spector MS, Kramer M, McCombie WR (2011). A comparative analysis of exome capture. Genome Biol.

[b32] Potato Genome Sequencing Consortium (2011). Genome sequence and analysis of the tuber crop potato. Nature.

[b33] Rauscher GM, Smart CD, Simko I, Bonierbale M, Mayton H, Greenland A, Fry WE (2006). Characterization and mapping of Rpi-ber, a novel potato late blight resistance gene from Solanum berthaultii. Theor. Appl. Genet.

[b34] Saintenac C, Faure S, Remay A, Choulet F, Ravel C, Paux E, Balfourier F, Feuillet C, Sourdille P (2011). Variation in crossover rates across a 3-Mb contig of bread wheat (Triticum aestivum) reveals the presence of a meiotic recombination hotspot. Chromosoma.

[b35] Schornack S, Ballvora A, Gürlebeck D, Peart J, Baulcombe D, Ganal M, Baker B, Bonas U, Lahaye T (2004). The tomato resistance protein Bs4 is a predicted non-nuclear TIR-NB-LRR protein that mediates defense responses to severely truncated derivatives of AvrBs4 and overexpressed AvrBs3. Plant J.

[b36] Schulze-Lefert P, Panstruga R (2011). A molecular evolutionary concept connecting nonhost resistance, pathogen host range, and pathogen speciation. Trends Plant Sci.

[b37] Śliwka J, Jakuczun H, Chmielarz M, Hara-Skrzypiec A, Tomczyńska I, Kilian A, Zimnoch-Guzowska E (2012). Late blight resistance gene from Solanum ruiz-ceballosii is located on potato chromosome X and linked to violet flower colour. BMC Genet.

[b38] Tai TH, Dahlbeck D, Clark ET, Gajiwala P, Pasion R, Whalen MC, Stall RE, Staskawicz BJ (1999). Expression of the Bs2 pepper gene confers resistance to bacterial spot disease in tomato. Proc. Natl Acad. Sci. U.S.A.

[b39] The Arabidopsis Genome Initiative (2000). Analysis of the genome sequence of the flowering plant Arabidopsis thaliana. Nature.

[b40] Tomato Genome Consortium (2012). The tomato genome sequence provides insights into fleshy fruit evolution. Nature.

[b41] Ooijen Van JW (2006).

[b42] Weigel DGJ (2002). How to isolate a gene defined by a mutation. In Arabidopsis. A Laboratory Manual.

[b43] Yandell M, Ence D (2012). A beginner's guide to eukaryotic genome annotation. Nat. Rev. Genet.

[b44] Zerbino DR, Birney E (2008). Velvet: algorithms for de novo short read assembly using de Bruijn graphs. Genome Res.

[b45] Zhang GY, Chen M, Guo JM, Xu TW, Li LC, Xu ZS, Ma YZ, Chen XP (2009). Isolation and characteristics of the CN gene, a tobacco mosaic virus resistance N gene homolog, from tobacco. Biochem. Genet.

