# Supplementary material for: Resistance gene enrichment sequencing (RenSeq) enables reannotation of the NB-LRR gene family from sequenced plant genomes and rapid mapping of resistance loci in segregating populations
Source: Plant J. 2013 Oct 8;76(3):530–44. doi: 10.1111/tpj.12307 (PMC3935411; doi:10.1111/tpj.12307)
Supplement: Figure S1 — Physical mapping position of all potato NB-LRR loci. [file tpj0076-0530-sd1.pdf]

Jupe and Witek et al.
Supporting Information

Figure S1. Physical mapping position of all potato NB-LRR loci.
All NB-LRR loci were mapped onto the twelve chromosomes of the reference potato clone DM. The underlying color code is as in (Jupe et al. 2012), and the RenSeq based newly identified NB-LRR loci are shown in purple. The chromosomes and locations are drawn to scale.

DM NB-LRR genes

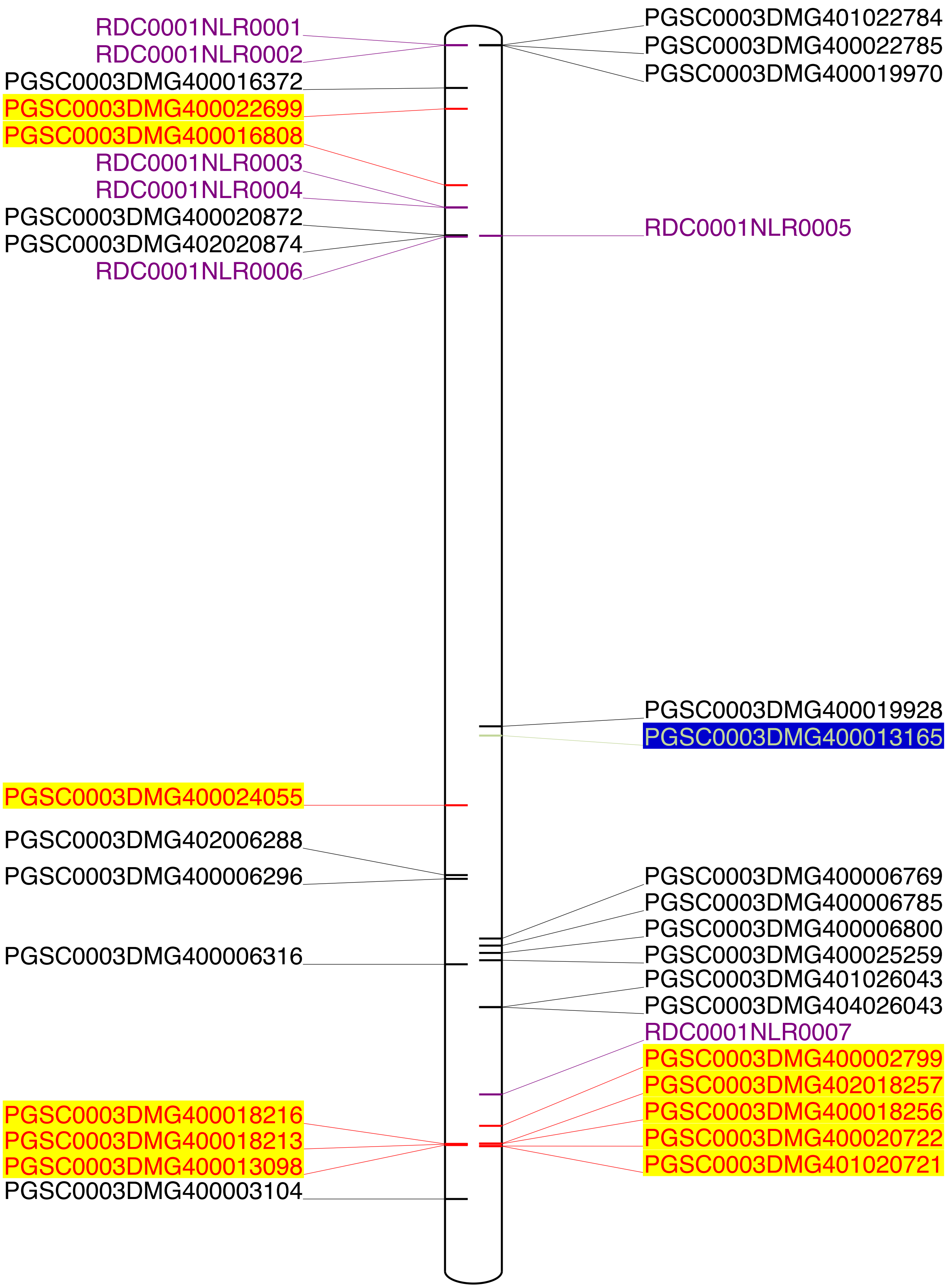

Chromosome 01

DM NB-LRR genes

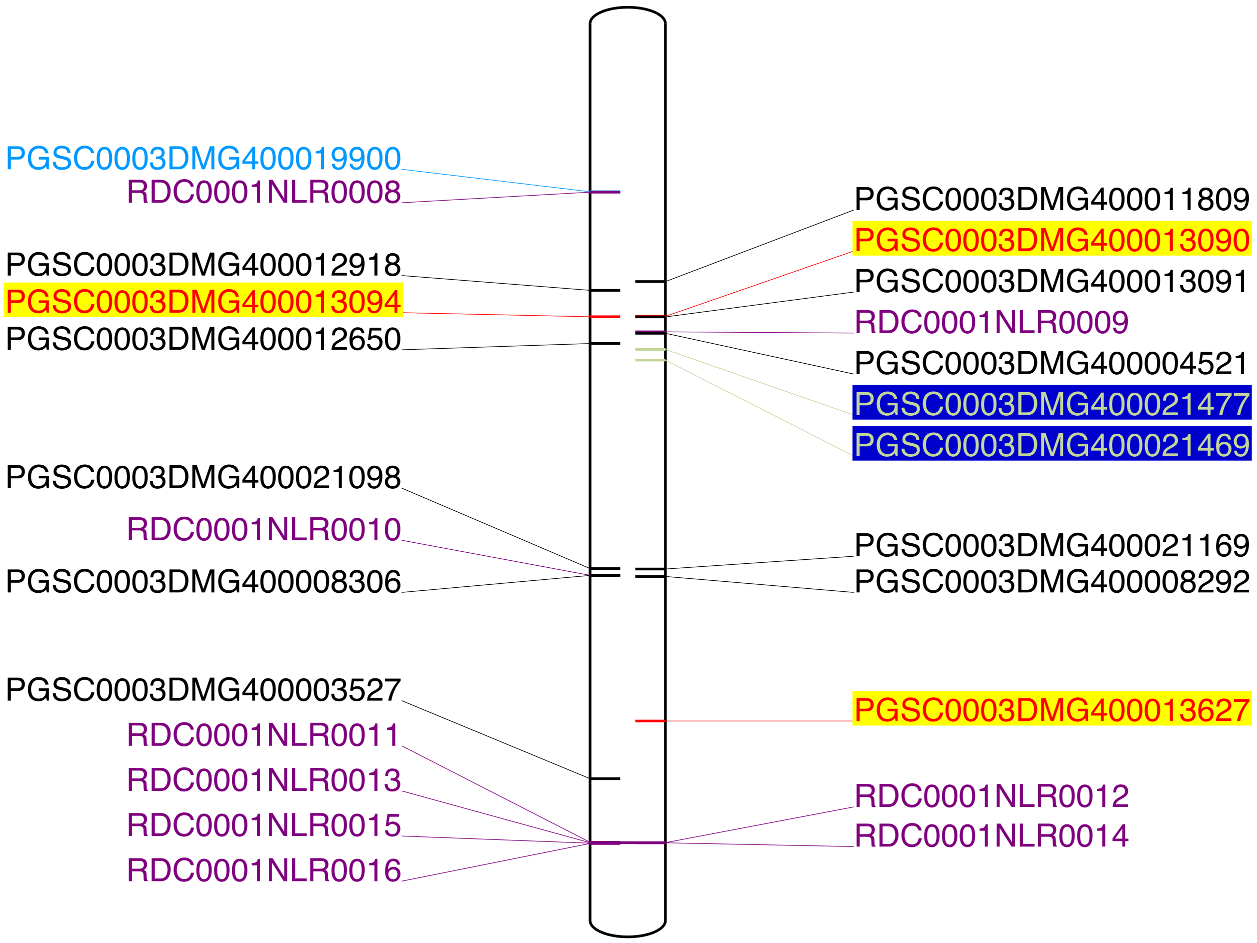

Chromosome 02

DM NB-LRR genes

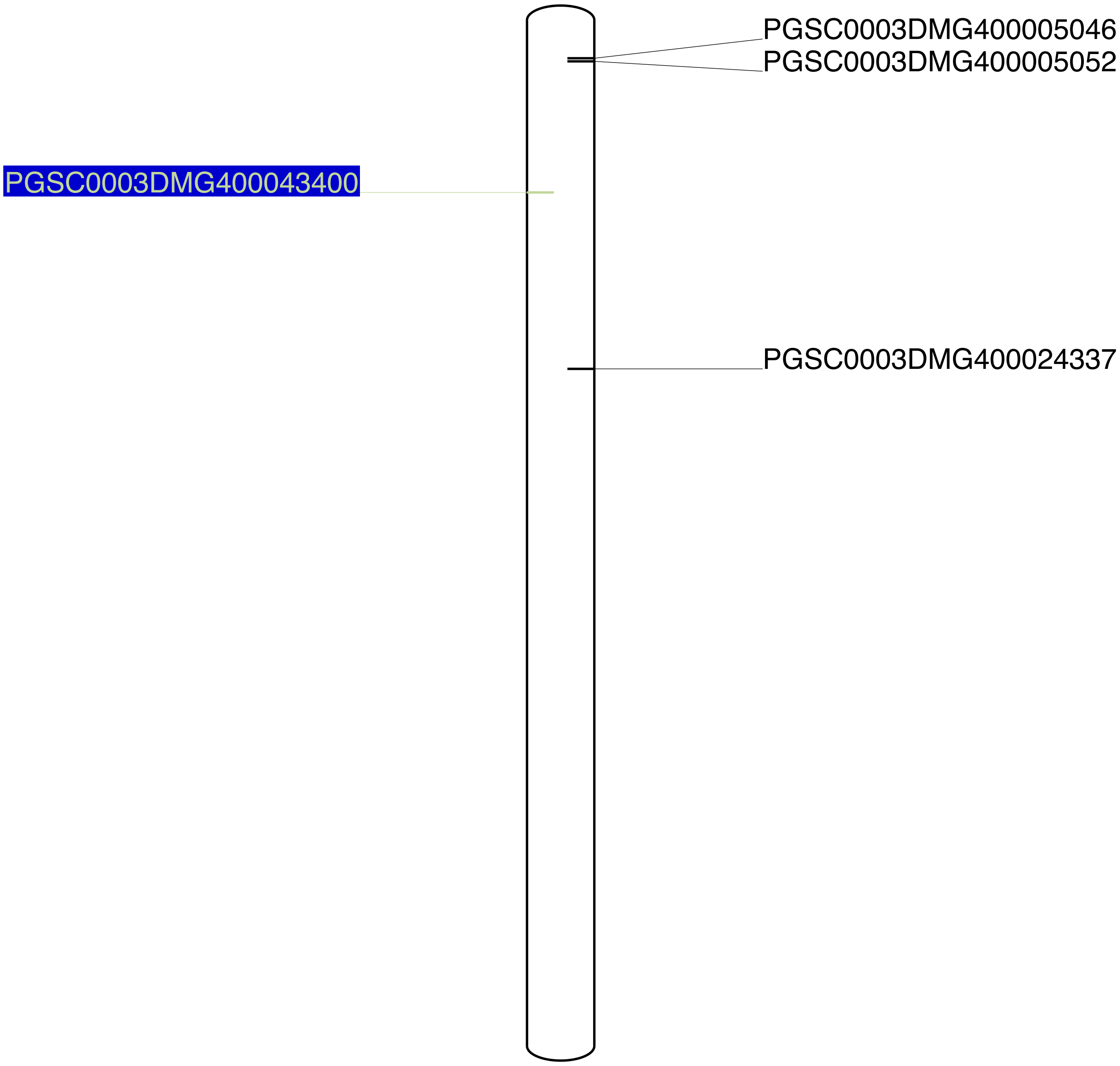

Chromosome 03

DM NB-LRR genes

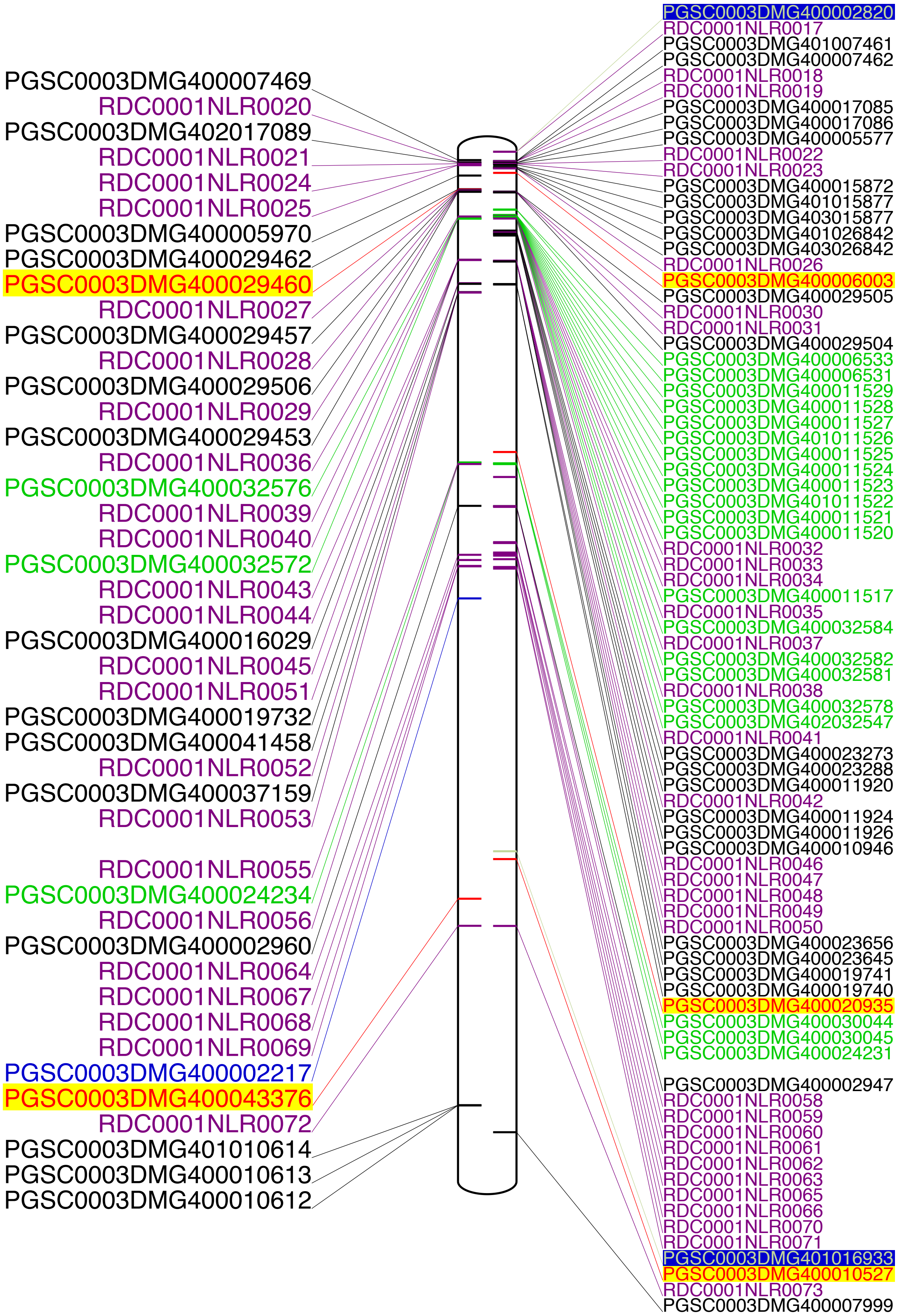

Chromosome 04

DM NB-LRR genes

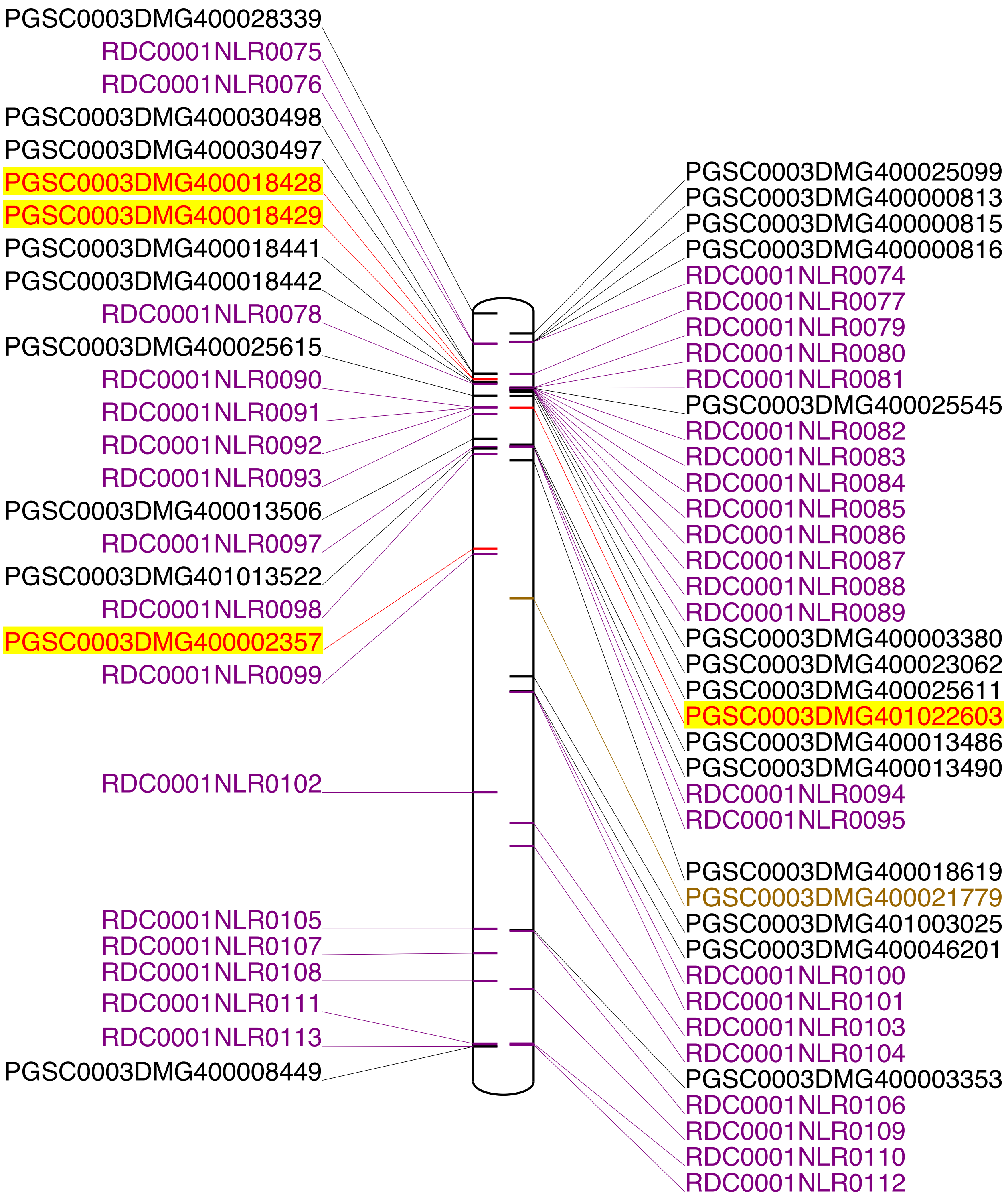

Chromosome 05

DM NB-LRR genes

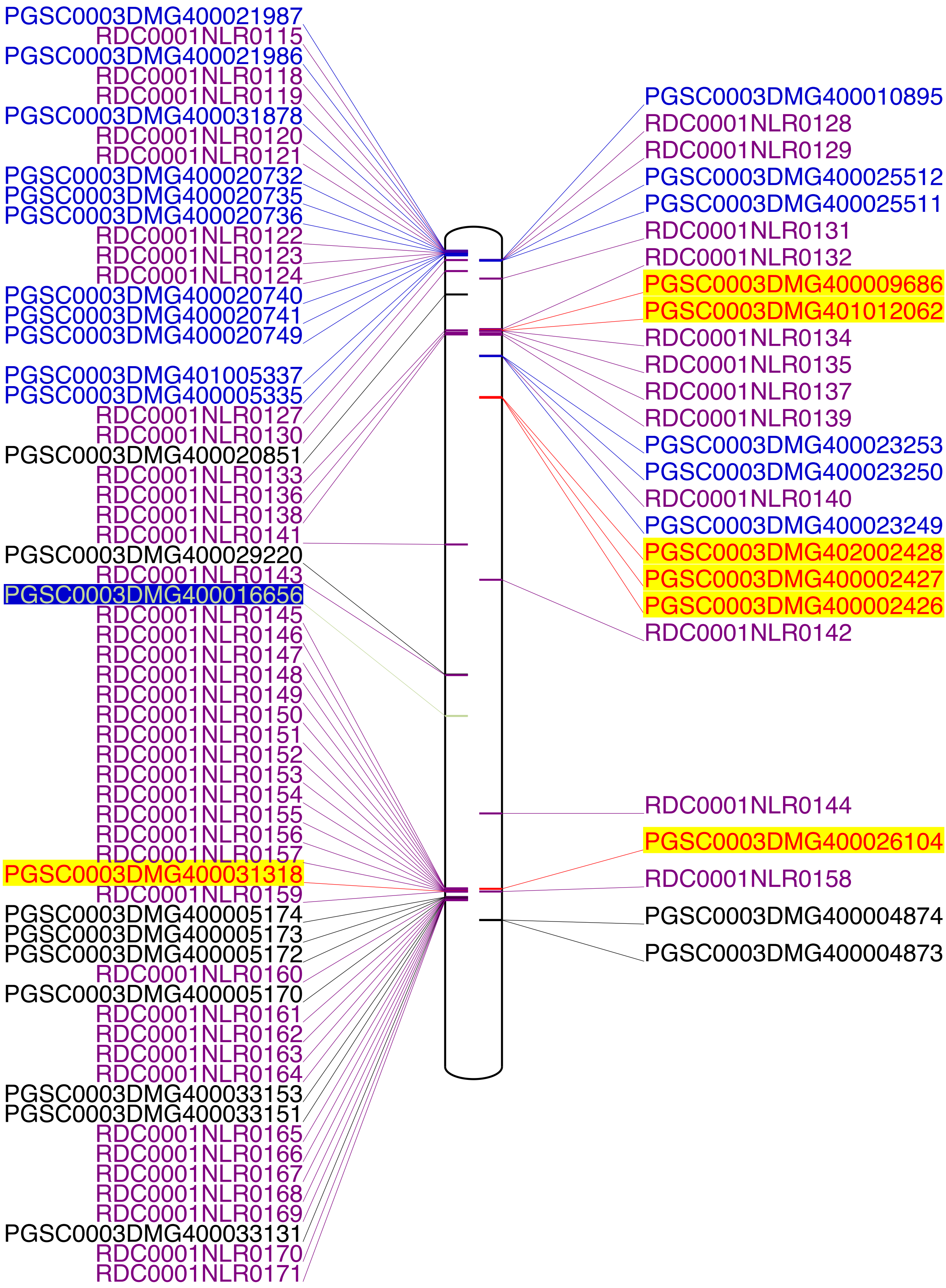

Chromosome 06

DM NB-LRR genes

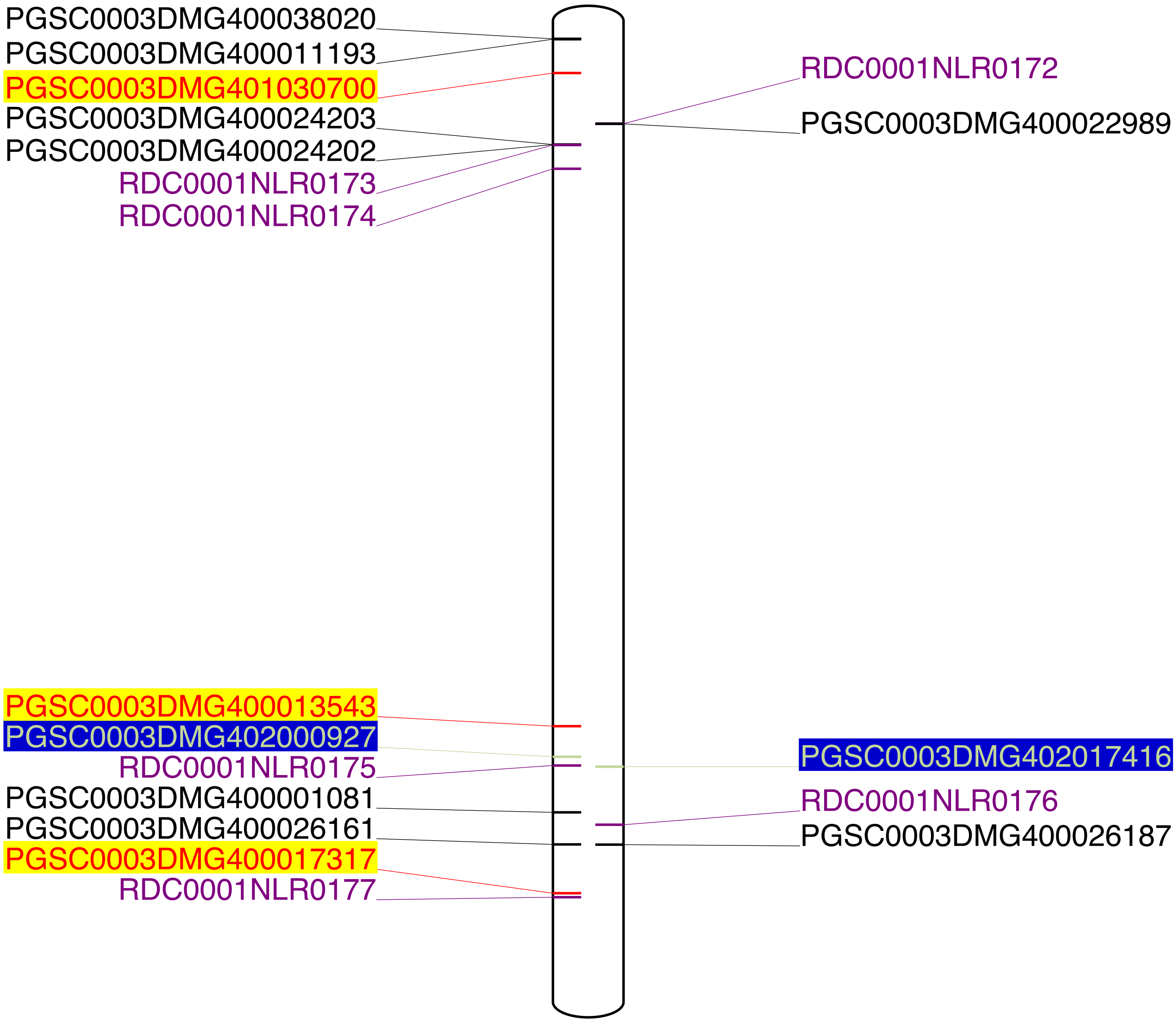

Chromosome 07

DM NB-LRR genes

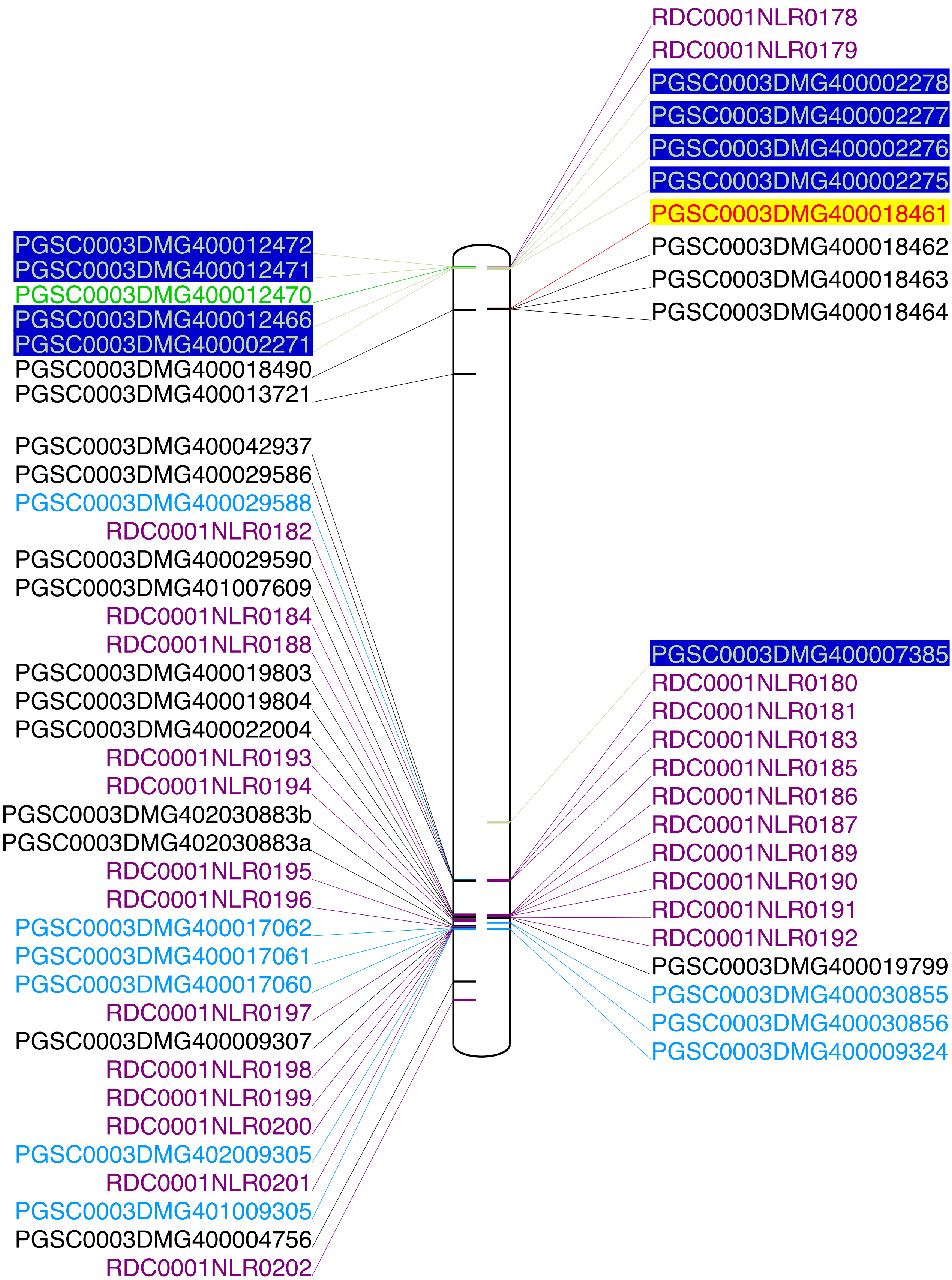

Chromosome 08

DM NB-LRR genes

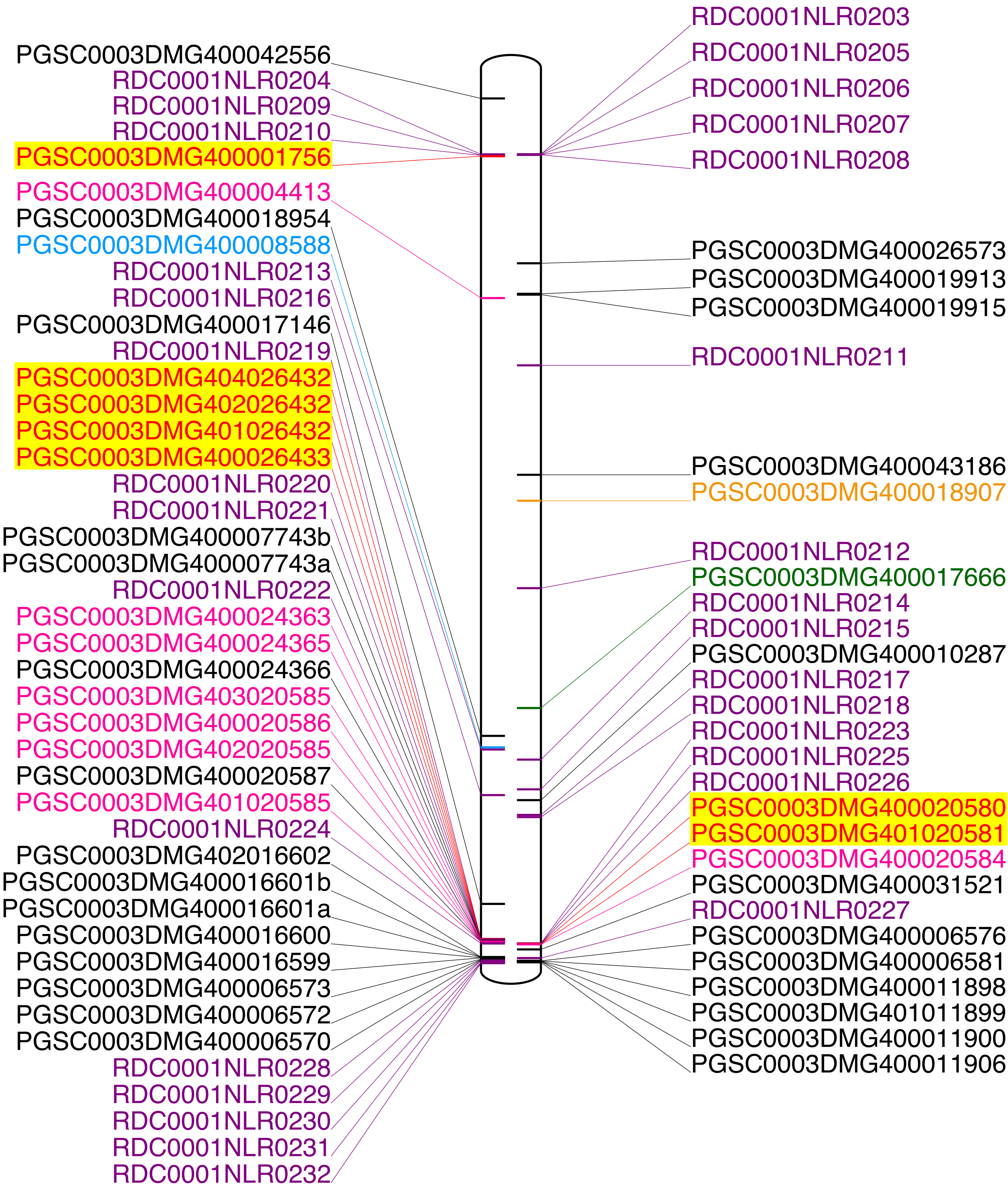

Chromosome 09

DM NB-LRR genes

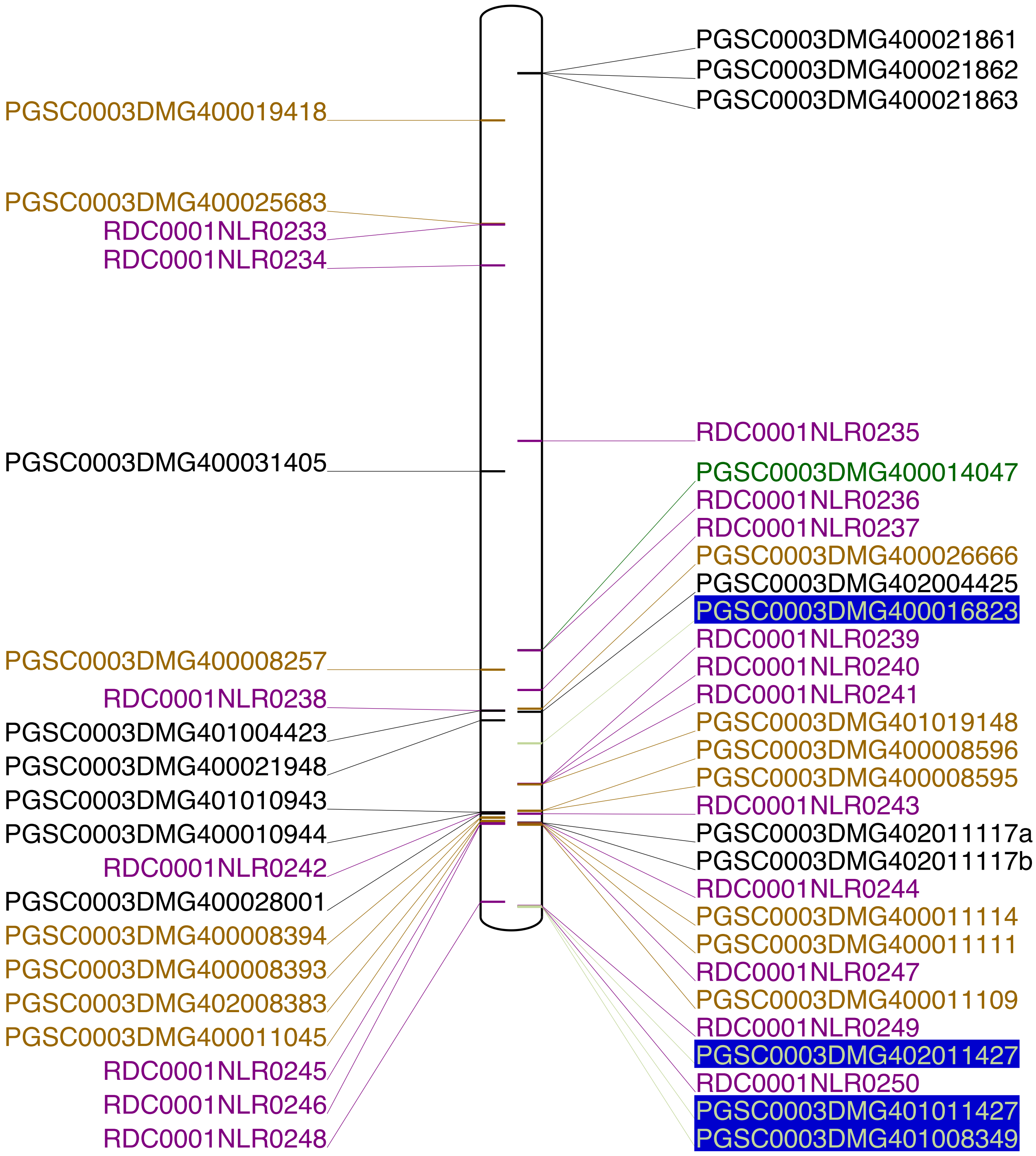

Chromosome 10

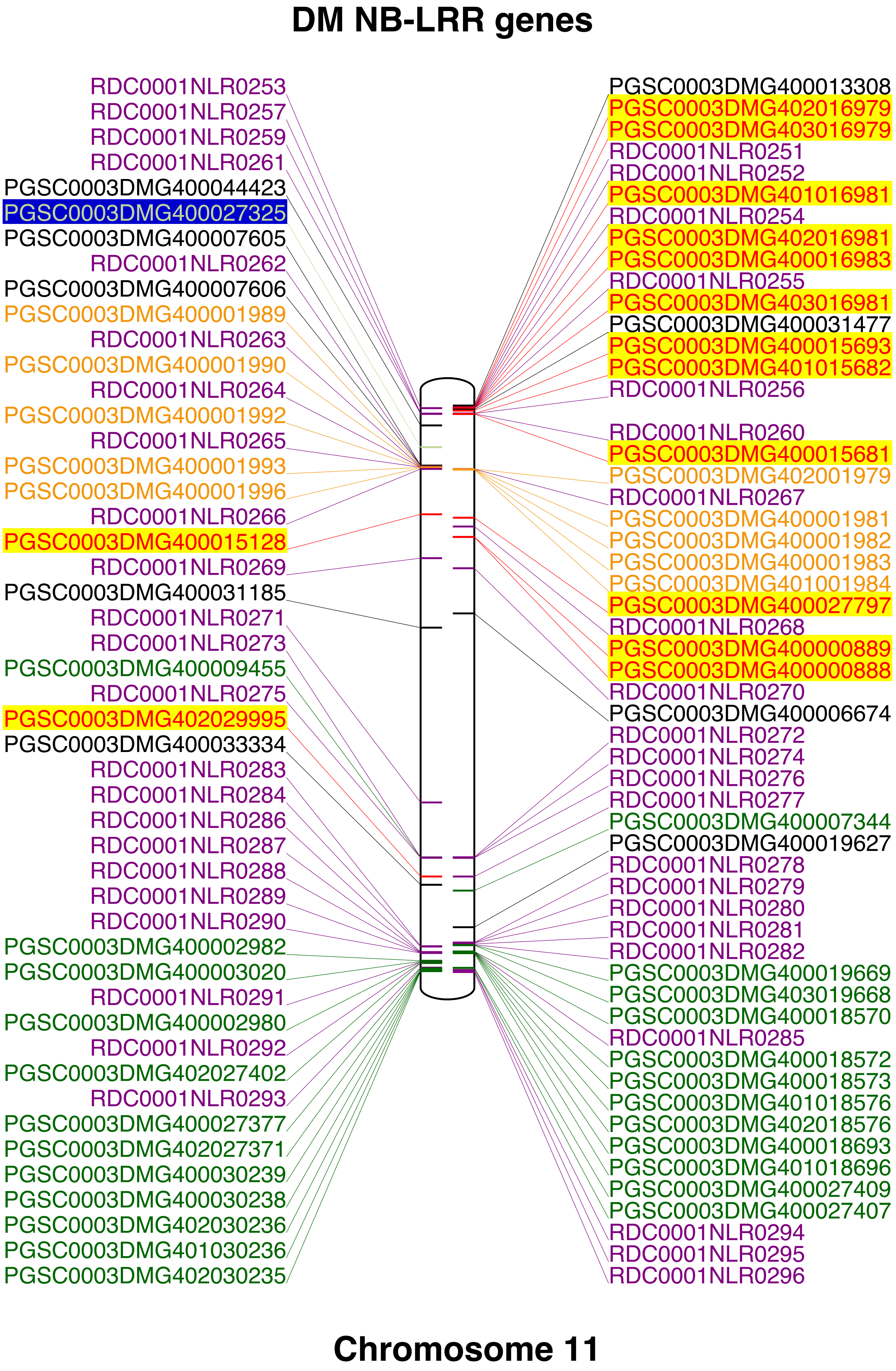
DM NB-LRR genes
RDC0001NLR0253
RDC0001NLR0257
RDC0001NLR0259
RDC0001NLR0261
PGSC0003DMG400044423
PGSC0003DMG400027325
PGSC0003DMG400007605
RDC0001NLR0262
PGSC0003DMG400007606
PGSC0003DMG400001989
RDC0001NLR0263
PGSC0003DMG400001990
RDC0001NLR0264
PGSC0003DMG400001992
RDC0001NLR0265
PGSC0003DMG400001993
PGSC0003DMG400001996
RDC0001NLR0266
PGSC0003DMG400015128
RDC0001NLR0269
PGSC0003DMG400031185
RDC0001NLR0271
RDC0001NLR0273
PGSC0003DMG400009455
RDC0001NLR0275
PGSC0003DMG402029995
PGSC0003DMG400033334
RDC0001NLR0283
RDC0001NLR0284
RDC0001NLR0286
RDC0001NLR0287
RDC0001NLR0288
RDC0001NLR0289
RDC0001NLR0290
PGSC0003DMG400002982
PGSC0003DMG400003020
RDC0001NLR0291
PGSC0003DMG400002980
RDC0001NLR0292
PGSC0003DMG402027402
RDC0001NLR0293
PGSC0003DMG400027377
PGSC0003DMG402027371
PGSC0003DMG400030239
PGSC0003DMG400030238
PGSC0003DMG402030236
PGSC0003DMG401030236
PGSC0003DMG402030235
PGSC0003DMG400013308
PGSC0003DMG402016979
PGSC0003DMG403016979
RDC0001NLR0251
RDC0001NLR0252
PGSC0003DMG401016981
RDC0001NLR0254
PGSC0003DMG402016981
PGSC0003DMG400016983
RDC0001NLR0255
PGSC0003DMG403016981
PGSC0003DMG400031477
PGSC0003DMG400015693
PGSC0003DMG401015682
RDC0001NLR0256
RDC0001NLR0260
PGSC0003DMG400015681
PGSC0003DMG402001979
RDC0001NLR0267
PGSC0003DMG400001981
PGSC0003DMG400001982
PGSC0003DMG400001983
PGSC0003DMG401001984
PGSC0003DMG400027797
RDC0001NLR0268
PGSC0003DMG400000889
PGSC0003DMG400000888
RDC0001NLR0270
PGSC0003DMG400006674
RDC0001NLR0272
RDC0001NLR0274
RDC0001NLR0276
RDC0001NLR0277
PGSC0003DMG400007344
PGSC0003DMG400019627
RDC0001NLR0278
RDC0001NLR0279
RDC0001NLR0280
RDC0001NLR0281
RDC0001NLR0282
PGSC0003DMG400019669
PGSC0003DMG403019668
PGSC0003DMG400018570
RDC0001NLR0285
PGSC0003DMG400018572
PGSC0003DMG400018573
PGSC0003DMG401018576
PGSC0003DMG402018576
PGSC0003DMG400018693
PGSC0003DMG401018696
PGSC0003DMG400027409
PGSC0003DMG400027407
RDC0001NLR0294
RDC0001NLR0295
RDC0001NLR0296
Chromosome 11

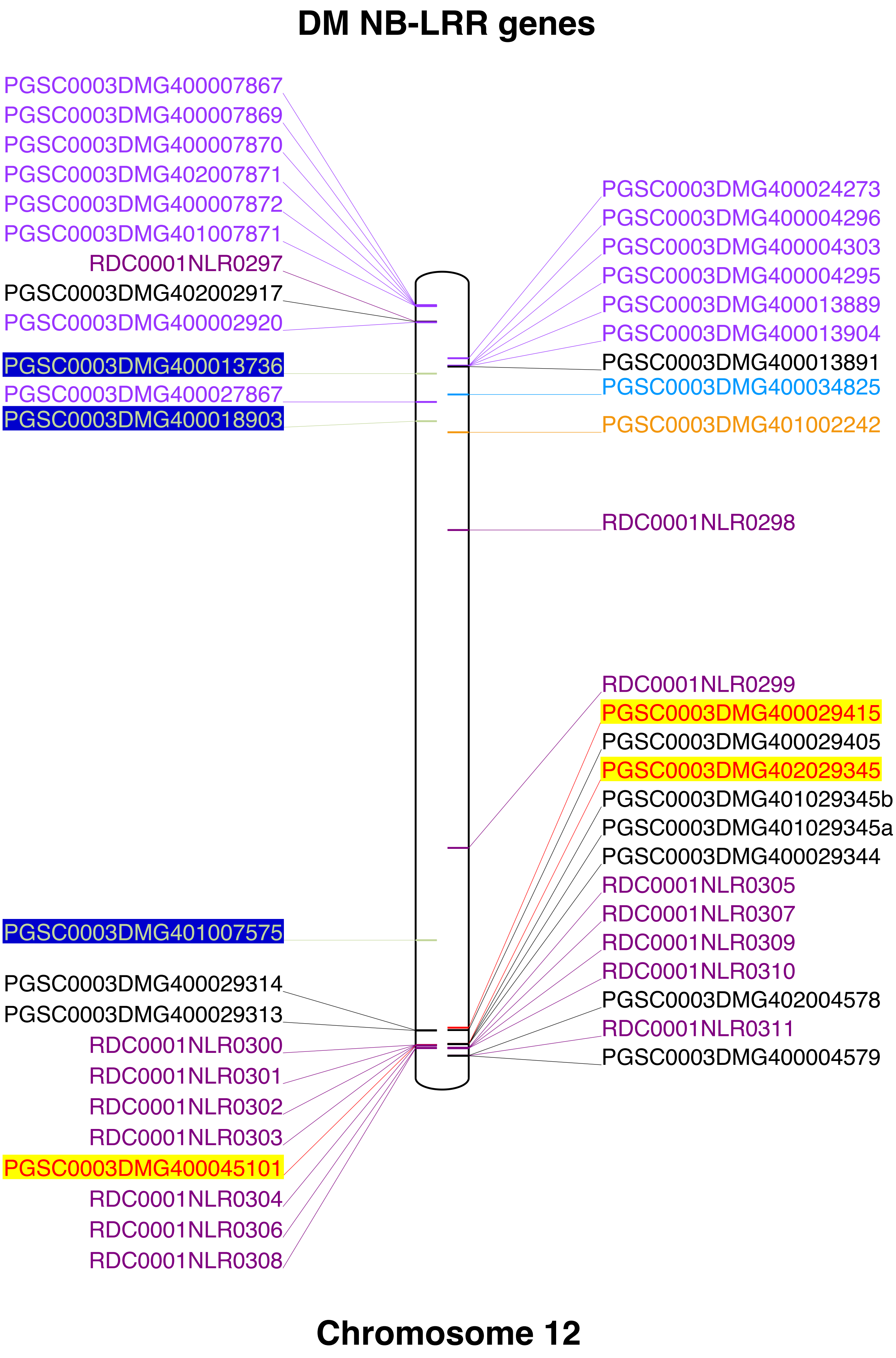
DM NB-LRR genes
PGSC0003DMG400007867
PGSC0003DMG400007869
PGSC0003DMG400007870
PGSC0003DMG402007871
PGSC0003DMG400007872
PGSC0003DMG401007871
RDC0001NLR0297
PGSC0003DMG402002917
PGSC0003DMG400002920
PGSC0003DMG400013736
PGSC0003DMG400027867
PGSC0003DMG400018903
PGSC0003DMG401007575
PGSC0003DMG400029314
PGSC0003DMG400029313
RDC0001NLR0300
RDC0001NLR0301
RDC0001NLR0302
RDC0001NLR0303
PGSC0003DMG400045101
RDC0001NLR0304
RDC0001NLR0306
RDC0001NLR0308
PGSC0003DMG400024273
PGSC0003DMG400004296
PGSC0003DMG400004303
PGSC0003DMG400004295
PGSC0003DMG400013889
PGSC0003DMG400013904
PGSC0003DMG400013891
PGSC0003DMG400034825
PGSC0003DMG401002242
RDC0001NLR0298
RDC0001NLR0299
PGSC0003DMG400029415
PGSC0003DMG400029405
PGSC0003DMG402029345
PGSC0003DMG401029345b
PGSC0003DMG401029345a
PGSC0003DMG400029344
RDC0001NLR0305
RDC0001NLR0307
RDC0001NLR0309
RDC0001NLR0310
PGSC0003DMG402004578
RDC0001NLR0311
PGSC0003DMG400004579
Chromosome 12
