# Supplementary figures and images for: Resistance gene enrichment sequencing (RenSeq) enables reannotation of the NB-LRR gene family from sequenced plant genomes and rapid mapping of resistance loci in segregating populations
Source: Plant J. 2013 Oct 8;76(3):530–44. doi: 10.1111/tpj.12307 (PMC3935411; doi:10.1111/tpj.12307)

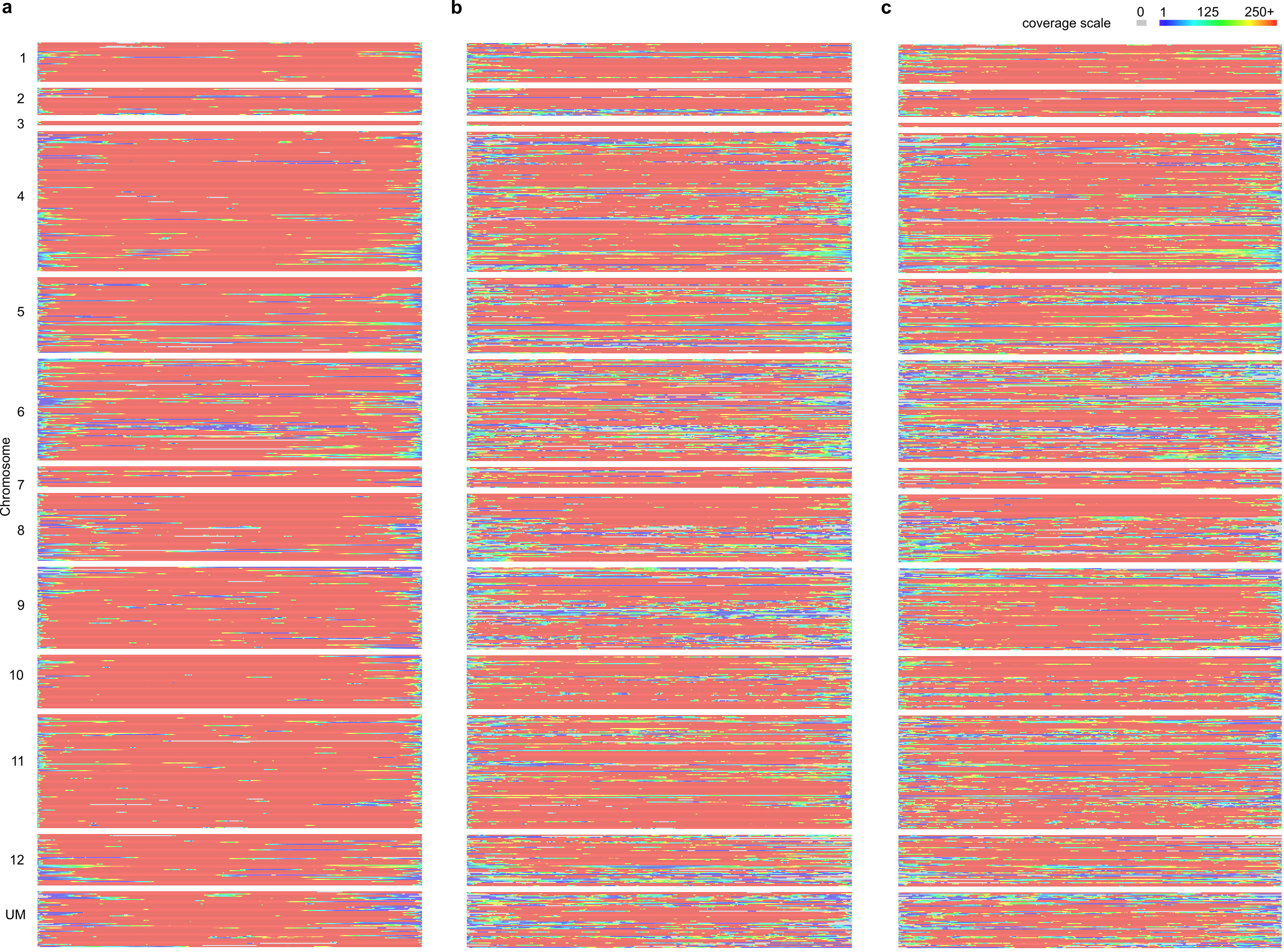

Supplement: Figure S2 — Visualisation of NB-LRR read coverage after RenSeq of DM, Rpi-ber2 and Rpi-rzc1. [file tpj0076-0530-sd2.jpg]

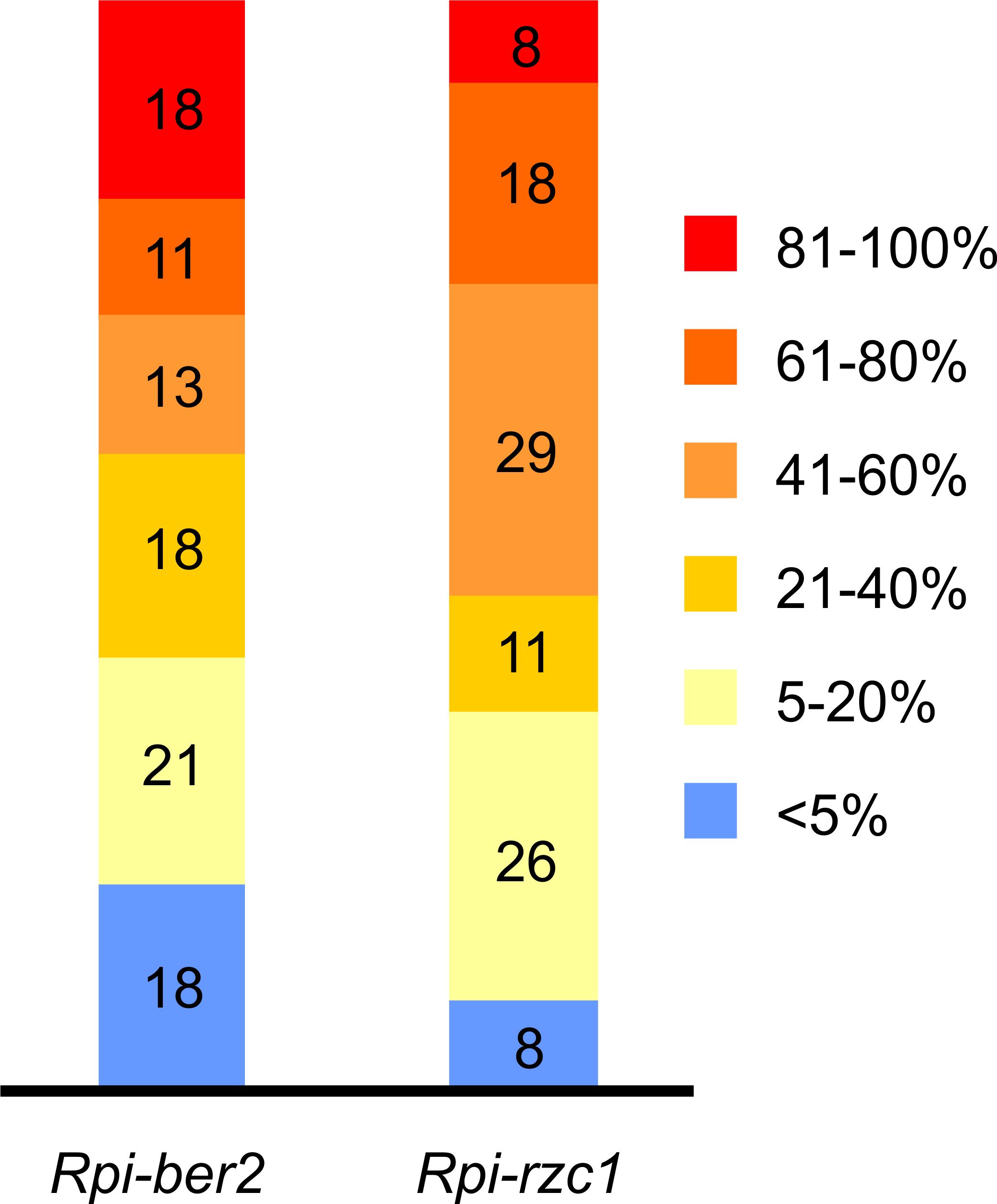

Supplement: Figure S3 — The coverage of chromosome 10 DM NB-LRRs with de novo assembled contigs from Rpi-ber2 and Rpi-rzc1. [file tpj0076-0530-sd3.jpg]
