# Supplementary material for: Resistance gene enrichment sequencing (RenSeq) enables reannotation of the NB-LRR gene family from sequenced plant genomes and rapid mapping of resistance loci in segregating populations
Source: Plant J. 2013 Oct 8;76(3):530–44. doi: 10.1111/tpj.12307 (PMC3935411; doi:10.1111/tpj.12307)
Supplement: Methods S1 — A detailed section containing all experimental procedures. [file tpj0076-0530-sd13.docx]

**Supplementary Methods S1**

**Design of a customized NB-LRR enrichment library**

Capture of NB-LRR like sequences was carried out using a solution based customized Agilent SureSelect Target Enrichment kit (Agilent Technologies, Inc.). A first draft of the potato NB-LRR gene complement was established in 2009 using two different strategies. The first strategy is similar to Jupe et al. (2012) but was carried out on version 3.0 of the sequenced potato genome annotation. The second strategy identified putative NB-LRR gene sequences through Pfam-domain searches (pfam.sanger.ac.uk) and further hmm-models in the predicted coding-sequences of the same genome annotation. In addition, a list of NB-ARC domain containing sequences was generated from tomato using the previously applied hmm-model. A blastx (Altschul et al. 1990) search against the nr-database of NCBI confirmed sequence similarity to NB-LRR type *R* gene homologs.

The bait-oligos were designed over and between the exon-exon boundaries of the retrieved sequences. The first bait started at the left most nucleotide and followed the predicted coding direction of the gene. Target sequences were at least two times tiled with 60nt overlap, and bait sequences containing Ns were removed. In order to utilize the full capacity of around 55,000 baits, oligos derived from NB-ARC domains were duplicated. In addition, nine characterized Solanaceae NB-LRR type *R* genes were included: *Bs2* (NCBI-accession AF202179, Tai et al. 1999), *Bs4* AY438027 (Schornack et al. 2004), *Hero* AJ457051 (Ernst et al. 2002), *I2* AF118127 (Ori et al. 1997), *Mi1* AF039682 (Milligan et al. 1998), *N* EF091690 (Zhang et al. 2009), *Sw-5* (Brommonschenkel et al. 2000), *Tm-1* NM_001247615 (Ishibashi et al. 2007) and *Tm-2* EF137705 (Lanfermeijer et al. 2003). In total 48,549 120-mer biotinylated RNA baits were designed, and subsequently synthesized by Agilent Technologies Inc.

**Analysis of the bait-library**

To identify the maximum observed distance between bait and sequence, 100,000 representative FASTA-formatted reads were randomly selected from a large dataset of NB-LRR enriched Illumina reads from several studies (DM, *Rpi-rzc1*, *Rpi-ber2*, *S. michoacanum* and *S. nigrum*). Sequence similarity searches using blastn were carried out using the bait-library as the blast database and the reads as query sequences (default parameters). Using the blast output file, the sequence identity to the top hit was noted for each query and a distribution of sequence identities between the reads and the bait-library was produced. This distribution provides a description of the maximum distance between the reads and the bait-library in relation to the experimental parameters used to create the reads, i.e. how dissimilar DNA fragments can be to the bait and still bind.

The bait-library was mapped against the tomato chromosomes S_lycopersicum_chromosomes.2.40.fa individually, using the Geneious tool “*Map to Reference*” (map multiple best matches, maximum mismatch 20%, word length 6). A set of ten gene models annotated as NB-LRR genes and manually confirmed by us, were used to determine the coverage. Subsequently all regions with a coverage of eight baits or higher over at least 200nt were extracted including approximately 1-2kb flanking region and analyzed in a blastn sequence search against the DM NB-LRR genes or the NCBI nr-database. Single gene models that belong to a longer sequence were analyzed as described in (Jupe et al. 2012). Positions of regions harboring NB-LRR like sequences were compared to all tomato gene models to identify novel sequences and wrongly annotated genes.

**Target capture**

Target capture was carried out according to the protocol of Agilent with minor modifications. Briefly, a total of 1µg gDNA was fragmented for 30s, 20% duty cycle, intensity level 5, and 200 cycles/burst (to get ~500 nt fragments) (High-Performance Ultra-Sonicator, Covaris, Inc.). Sheared gDNA was purified using 1.8× AMPure XP beads (BECKMAN COULTER) and further processed for Illumina paired-end sequencing using the NEBNext DNA Sample Prep Reagent Set 1 according to the manufacturer’s protocol (NEW ENGLAND BioLabs Inc.). After each step the fragment size was checked by electrophoresis on a 1.5% agarose gel and all fragments were purified using 1:1 (v:v) AMPure XP beads (BECKMAN COULTER). DNA fragments were end-repaired, A-overhangs added and Illumina paired-end sequencing adapters were ligated to them. A PCR was carried out using Herculase II polymerase (Agilent Technologies, Inc.) with 10µl DNA template and primers annealing to Illumina PE adaptors in a 50µl reaction mix. Visualization on an electrophoresis gel showed sufficient amplification. Amplified DNA was combined with the hybridization block-mix containing 5µl plant capture enhancer (Roche Diagnostics Ltd) and 0.6µl Hyb-Block #3 (Agilent Technologies, Inc.). Hybridization to the custom RNA-baits took place in one half of the recommended reaction volume (14μl) for 22h at 65°C in a thermal cycler. RNA-DNA hybrids were selected using Dynal magnetic beads (Invitrogen, Life Technologies Corporation), purified with AMPure XP beads and eluted in 15µl Sigma water (SIGMA-ALDRICH Co. LLC.). Post-capture PCR (Herculase II polymerase) was carried out in 50µl with 15µl template. PCR products were purified with AMPure XP beads, eluted in 15µl Sigma water and measured using Quant-iT PicoGreen (Invitrogen Ltd) according to the manufacturer’s recommendations. All DNA libraries were size selected (fragments of around 600nt) by cutting from polyacrylamide gel.

Enrichment efficacy was assessed in a quantitative PCR SYBR Green assay (Sigma-Aldrich Co.) with 1ng starting material from enriched and non-enriched samples, 0.5µl of the corresponding NB-ARC specific primer (100nM final concentration) and 8µl water (50 cycles in qPCR). Primer information is disclosed in Table S7.

**Illumina sequencing and raw sequence data processing**

All quality control, mapping and sequence assembly experiments of Illumina paired-end sequences were carried out using tools embedded in The Sainsbury Laboratory (TSL) customized Galaxy instance (Giardine et al. 2005; Blankenberg et al. 2010; Goecks et al. 2010; Maclean and Kamoun 2012), if not noted otherwise. Raw Illumina paired-end reads were converted into Sanger FASTQ-format and quality controlled after joining the read pairs. Joint reads shorter than 152nt, containing N, and/or having a PHRED quality score below 20 (allowing for 35 bases outside this range) were removed. Reads were then split and mapped using BWA (Li and Durban 2009) against the required reference using default parameters, but allowing for two mismatches in the seed and four in the extension. The read coverage over off-target genes (as defined in Jupe et al. 2012) was assessed by stringently mapping the left-hand reads to the negative training set. Only left-hand reads were used to reduce the amount of computing resources. The conditions allowed for two mismatches in the extension, while other settings were default.

To count the number of reads mapping to any specific reference we used SAM-tools (Li and Durban 2009).

Genomic data was visualized with the genome browser function of Geneious (Geneious Pro 5.6, Biomatters; available from <http://www.geneious.com/>) and Savant Genome Browser (Fiume et al. 2012). A gff-format genome annotation file, which included all the DM NB-LRR gene positions, was created manually and imported into Savant/Geneious in order to view the NB-LRR loci on the genome.

Blast (Altschul et al. 1990) searches were generally carried out using default parameters, either as local stand-alone Blast 2.2.24, embedded in Geneious Pro 5.6, or using NCBI BLAST+ in Galaxy.
