# Supplementary material for: Resistance gene enrichment sequencing (RenSeq) enables reannotation of the NB-LRR gene family from sequenced plant genomes and rapid mapping of resistance loci in segregating populations
Source: Plant J. 2013 Oct 8;76(3):530–44. doi: 10.1111/tpj.12307 (PMC3935411; doi:10.1111/tpj.12307)
Supplement: Supplementary file 18 [file tpj0076-0530-sd18.docx]

**Supporting Information**

Figure S1

Physical mapping position of all potato NB-LRR loci.

Figure S2

Visualisation of NB-LRR read coverage after RenSeq of DM, *Rpi-ber2* and *Rpi-rzc1.*

Figure S3

The coverage of chromosome 10 DM NB-LRRs with *de novo* assembled contigs from *Rpi-ber2* and *Rpi-rzc1*.

Table S1

Assessment of NB-LRR enrichment efficiency using quantitative PCR.

Table S2

List of identified DM potato NB-LRR loci including position, strand and MAST search result.

Table S3

Number of clustered NB-LRR loci and number of clusters per chromosome.

Table S4

List of identified tomato NB-LRR loci.

Table S5

Statistics of Velvet *de novo* assembly of RenSeq reads.

Table S6

Primer list and Sanger sequencing results for markers designed over potential polymorphisms that co-segregate with resistance.

Table S7

Primer sequences used in a qPCR to study the enrichment efficiency of NB-LRR sequences.

Data S1

The bait-library sequences.

Data S2

A FASTA-file for the 755 DM NB-LRR loci sequences.

Methods S1

A detailed section containing all experimental procedures.

Methods S2

Galaxy workflow file for the ‘quick mapping’ approach designed in this study.

Methods S3

Galaxy workflow file for the ‘genotype-specific mapping’ approach designed in this study.
